# Default polyfunctional T helper 1 response to ample signal 1 alone

**DOI:** 10.1038/s41423-020-0415-x

**Published:** 2020-04-20

**Authors:** Luca Danelli, Georgina Cornish, Julia Merkenschlager, George Kassiotis

**Affiliations:** 1 Retroviral Immunology, The Francis Crick Institute, 1 Midland Road, London, NW1 1AT UK; 2 Department of Medicine, Faculty of Medicine, Imperial College London, London, W2 1PG UK; 3 Present Address: Laboratory of Molecular Immunology, The Rockefeller University, New York, NY 10065 USA

**Keywords:** CD4 T cell priming, T helper differentiation, peptide-MHC II complex, Antigen presentation, Lymphocyte activation, Lymphocyte differentiation

## Abstract

CD4^+^ T cells integrate well-defined signals from the T-cell receptor (TCR) (signal 1) and a host of costimulatory molecules (signal 2) to initiate clonal expansion and differentiation into diverse functional T helper (Th) subsets. However, our ability to guide the expansion of context-appropriate Th subsets by deploying these signals in vaccination remains limited. Using cell-based vaccines, we selectively amplified signal 1 by exclusive presentation of an optimized peptide:MHC II (pMHC II) complex in the absence of classic costimulation. Contrary to expectations, amplified signal 1 alone was strongly immunogenic and selectively expanded high-affinity TCR clonotypes, despite delivering intense TCR signals. In contrast to natural infection or standard vaccines, amplified signal 1, presented by a variety of professional and nonprofessional antigen-presenting cells (APCs), induced exclusively polyfunctional Th1 effector and memory cells, which protected against retroviral infection and tumor challenge, and expanded tumor-reactive CD4^+^ T cells otherwise rendered unresponsive in tumor-bearing hosts. Together, our findings uncover a default Th1 response to ample signal 1 and offer a means to selectively prime such protective responses by vaccination.

## Introduction

Adaptive immunity and immunological memory rely on clonal expansion and differentiation of T cells bearing appropriate T-cell receptors (TCRs), which recognize cognate peptide:MHC (pMHC) complexes on the surface of antigen-presenting cells (APCs). T cells accumulate TCR signals, commonly referred to as signal 1, as a function of the magnitude and duration of pMHC engagement and commit to proliferation when a critical threshold is reached. However, the minimal pMHC requirements for T-cell activation are influenced by the availability of costimulation and are substantially higher for the priming of naïve T cells than of antigen-experienced T cells.^1,2^ This effect of costimulatory signals, collectively referred to as signal 2, is the basis of the two-signal hypothesis, whereby both signals 1 and 2 are required for T-cell activation.^3,4^

In addition to initiating clonal expansion, the overall strength of TCR signaling received by CD4^+^ T cells also influences functional differentiation into one or more distinguishable T helper (Th), follicular helper (Tfh) and regulatory T (Treg) cell subsets.^5–10^ Strong TCR signals are linked with Tfh differentiation, although this is not universally observed.^11–14^ Th differentiation is also guided by a multitude of T-cell-extrinsic factors that can override the influence of TCR signal strength to ensure appropriate Th subset development.^5–9^ This is a critical adaptation that allows CD4^+^ T cells of similar affinities to adopt distinct Th profiles, according to the immune context. However, Th differentiation is not always suitable to the requirements, and inappropriate Th subset development may compromise protective immunity or induce immune pathology. Indeed, the development of Th2, Th17 or Treg responses, instead of protective Th1 responses, can be detrimental in respiratory syncytial virus or *Mycobacterium tuberculosis* infections^15,16^ and in cancer.^17^ Despite detailed knowledge of the factors and transcriptional programs underpinning Th polarization,^5–9^ the ability to direct appropriate Th subset development is not yet fully developed in current vaccination or immunotherapy regimens.

An essential requirement for signal 2 for T-cell priming is thought to reflect the generally limiting availability of signal 1, the intensity of which is determined by the affinity of the TCR for the cognate pMHC and the abundance of such complexes on the surface of APCs. Both of these variables are limiting for T-cell activation, which requires engagement of several hundred or thousand TCRs^2^ by rare cognate pMHC complexes. Nevertheless, CD4^+^ T-cell activation can be triggered by as few as ~50–300 cognate pMHC II complexes among ~200,000 unrelated pMHC II complexes on each APC,^1,18,19^ and CD8^+^ T-cell cytotoxicity may be triggered by even fewer cognate pMHC I complexes,^20^ indicating that the effect of pMHC abundance on T-cell activation may not be linear. Moreover, instead of effective T-cell priming, overabundance of pMHC, particularly in the absence of signal 2, may lead to immunological tolerance through clonal deletion, anergy, exhaustion or immune suppression, as has been observed with ubiquitous self, transplantation or cancer antigens.^21–25^

While the precise contribution of accessory molecules might be context dependent, it has been well established that effective T-cell responses can be primed, boosted or invigorated by modulation of costimulatory and coinhibitory signals, in both experimental and clinical settings.^4,26^ However, modulation of T-cell responses by accessory signals bears no antigen specificity and affects T cells rather indiscriminately, which is often associated with immune pathology or autoimmunity.^26^ In contrast, modulation of T-cell responses by signal 1 availability prevents antigen nonspecific effects but has not been extensively applied owing to both gaps in our theoretical understanding of the T-cell responses to signal 1 alone and practical limitations in increasing pMHC abundance in vivo. This is particularly problematic in the case of MHC II-restricted peptides, where, when considering abundance, not all complexes carrying a given core peptide epitope are equivalent. MHC II-presented peptides often extend beyond the binding core, ranging from 7 to 35 amino acids in total.^27–29^ Core peptide-flanking residues (PFRs) vary in length and affect both the stability of pMHC complexes and recognition by the TCR by providing additional contact points with either the MHC II backbone or the TCR.^30,31^ Consequently, PFRs contribute to the ability of pMHC complexes to activate T cells, according to the particular combination of TCR and pMHC, and affect the clonotypic composition of the CD4^+^ T-cell response.^30,31^ T cells, therefore, likely encounter a range of cognate pMHC combinations of variable stimulatory capacity, depending on PFR length.

Here, we engineered an optimal pMHC II complex, which was presented to CD4^+^ T cells in high abundance by nonprofessional APCs, lacking classic costimulatory molecules. We show that, instead of CD4^+^ T-cell tolerance, priming with optimal signal 1 alone induced almost exclusively a highly functional Th1 effector and memory response compared with mixed Th responses induced by infection or standard immunization. Moreover, CD4^+^ T-cell memory induced by optimal signal 1 alone was significantly more protective against retroviral infection and tumor challenge than standard vaccines or immunization regimens, including natural infection and tumor challenge. Our data suggest that Th1 effector and memory development is the default CD4^+^ T-cell response to signal 1.

## Results

### Naïve CD4^+^ T-cell priming and memory formation by optimal signal 1 alone

To define priming requirements, we used a TCR transgenic system of the CD4^+^ T-cell response to the A^b^-restricted env_124–138_ epitope derived from the gp70 envelope glycoprotein of the Friend murine leukemia virus (F-MLV).^32,33^ Consistent with findings in other systems,^30,31^ PFR extensions of the env_124–138_ epitope affect the potency of stimulation of distinct TCR clonotypes^34^ and may consequently affect functional differentiation or other aspects of the CD4^+^ T-cell response. To determine whether env-reactive CD4^+^ T cells prefer an optimum peptide length, we tested the ability of nested env peptides, ranging in length from 11 to 35 amino acid residues (Fig. S1a), to stimulate EVα2 TCRαβ-transgenic CD4^+^ T cells.^33^ These monoclonal env-reactive CD4^+^ T cells were chosen to preclude PFR effects on different TCRα or β chains.^30,31,34^

As with the parental EF4.1 TCRβ-transgenic CD4^+^ T cells with the same reactivity,^34^ EVα2 T cells responded to these peptides according to the peptide dose and length, with the strongest upregulation of early activation markers, as well as commitment to proliferation, seen in the response to the highest doses of the 15-mer or the 20-mer (Fig. S1b). Late release of IFN-γ and IL-2 also peaked in response to the 15-mer or 20-mer, albeit at much lower concentrations of the peptides (Fig. S1b). These in vitro results indicated an optimal peptide length of approximately 17 amino acid residues, with shorter or longer peptides achieving only partial activation.

**Fig. 1:**
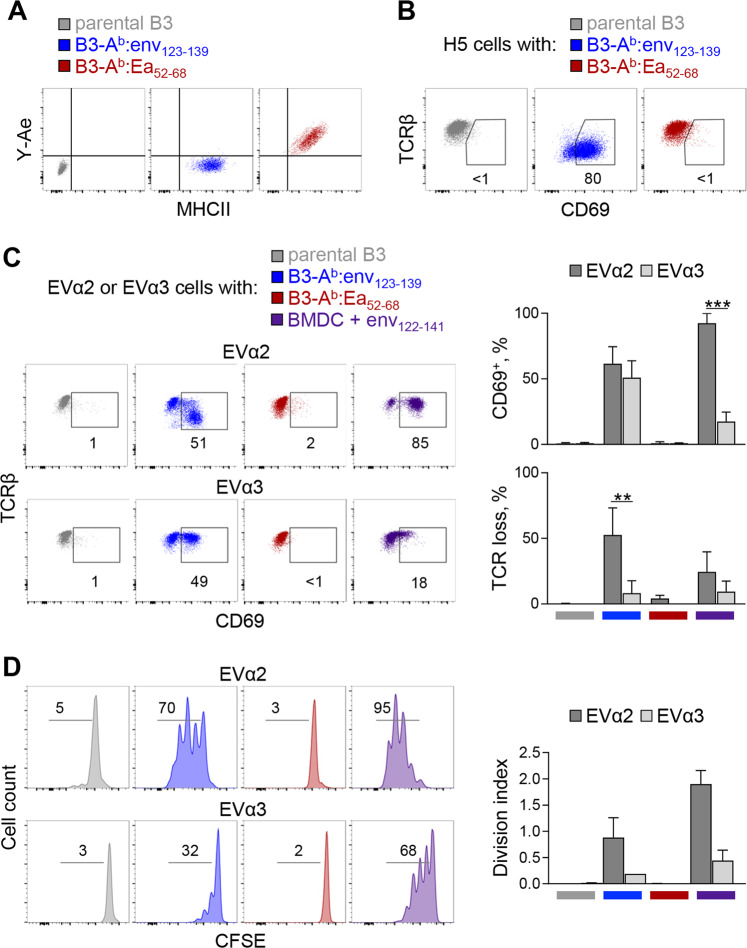
In vitro priming of naïve CD4^+^ T cells by optimal signal 1. **a** Expression of MHC II (detected with the M5/114.15.2 antibody) and specifically of A^b^:Ea_52−68_ complexes (detected with the Y-Ae antibody) in parental B3, B3-A^b^:env_123−139_ and B3-A^b^:Ea_52−68_ cells. **b** CD69 and TCRβ expression in env-specific H5 hybridoma cells after overnight culture with parental B3, B3-A^b^:env_123−139_ or B3-A^b^:Ea_52−68_ cells. **c** CD69 and TCRβ expression (*left*) and mean frequency (±SEM) (*right*) of primary EVα2 and EVα3 TCRαβ-transgenic CD4^+^ T cells upregulating CD69 or downregulating TCRβ 24 h after in vitro stimulation with parental B3, B3-A^b^:env_123−139_ or B3-A^b^:Ea_52−68_ cells or with env_122−141_ peptide-pulsed BM-DCs. **d** CFSE dilution (*left*) and calculated division index (±SEM) (*right*) of primary EVα2 and EVα3 TCRαβ-transgenic CD4^+^ T cells 72 h after in vitro stimulation with the same APCs as in **c**

Since presentation of a given peptide by MHC II molecules is typically limited by competition with numerous other peptides and by pMHC complex instability, we sought to overcome both of these limitations by covalently linking an env_123–139_ peptide with optimal length (17-mer) with the H2-A^b^ β chain. To ensure presentation of only the env_123–139_ peptide and dissociate the effects of cognate pMHC properties from those of additional signals and regulatory networks, we engineered nonprofessional APCs displaying exclusively A^b^:env_123–139_ complexes. To this end, B3 pro-B cell leukemia cells,^35^ lacking expression of MHC II and costimulatory molecules (Fig. S2), were transduced with retroviral vectors expressing H2-A^b^ α and β chains, with the latter covalently linked to the 17-mer env_123–139_ peptide (B3-A^b^:env_123–139_) or a control peptide from the H2-E^k^ α chain^27^ (B3-A^b^:Ea_52–68_) (Fig. 1a; Fig. S3). B3 cells expressing wild-type (WT) H2-A^b^ α and β chains (B3-A^b^), F-MLV gp70 (B3-gp70) or both (B3-A^b^;gp70) were also engineered as controls (Fig. S3).

Despite their immature state (Fig. S2), B3-A^b^:env_123–139_, but not parental B3 or control B3-A^b^:Ea_52–68_ cells, stimulated env-reactive T-cell hybridoma H5 cells very efficiently, as evidenced by TCR downregulation and CD69 upregulation (Fig. 1b). To test whether B3-A^b^:env_123–139_ cells could similarly stimulate naïve env-reactive T cells, we used high- and low-affinity EVα2 and EVα3 TCRαβ-transgenic CD4^+^ T cells, respectively.^33^ Expression of costimulatory molecules remained absent in B3 cells even after interaction with CD4^+^ T cells, with the exception of slight upregulation of OX40L (Fig. S4). In contrast to dendritic cells (DCs) pulsed with 10 µm env_122–141_ peptide, which preferentially stimulated the high-affinity EVα2 T cells, as expected,^33^ B3-A^b^:env_123–139_ cells stimulated comparable CD69 expression in both the EVα2 and EVα3 T cells in vitro (Fig. 1c). However, B3-A^b^:env_123–139_ cells induced more pronounced TCR downregulation than peptide-pulsed DCs in EVα2, but not EVα3, T cells (Fig. 1c). Moreover, despite the initial upregulation of CD69, EVα3 T cells failed to proliferate in response to B3-A^b^:env_123–139_ cells, in contrast to EVα2 T cells, which proliferated extensively (Fig. 1d). These results demonstrated that B3-A^b^:env_123–139_ cells delivered stronger TCR signals than DCs pulsed with 10 µm env_122–141_ peptide but did not abolish TCR affinity hierarchies.

**Fig. 2:**
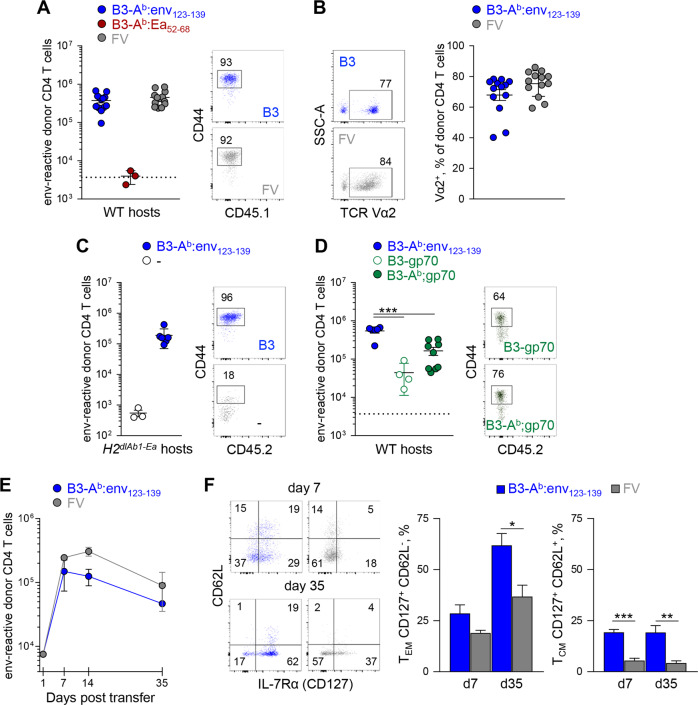
In vivo priming of naïve CD4^+^ T cells and memory formation by optimal signal 1. **a** Absolute numbers of (*left*) and CD44 expression (*right*) in donor TCRβ-transgenic EF4.1 CD4^+^ T cells 7 days after transfer into WT recipients and infection with FV or immunization with B3-A^b^:env_123−139_ or B3-A^b^:Ea_52−68_ cells. **b** TCR Vα2 expression (*left*) and frequency (±SEM) of Vα2^+^ cells (*right*) in donor EF4.1 CD4^+^ T cells 7 days after transfer into WT recipients and FV infection or B3-A^b^:env_123−139_ immunization. **c** Absolute numbers of (*left*) and CD44 expression (*right*) in donor EF4.1 CD4^+^ T cells 7 days after transfer into *H2*^*dlAb1-Ea*^ hosts, with or without B3-A^b^:env_123−139_ immunization. **d** Absolute numbers of (*left*) and CD44 expression (*right*) in donor EF4.1 CD4^+^ T cells 7 days after transfer into WT hosts and immunization with B3-A^b^:env_123−139_, B3-gp70 or B3-A^b^;gp70 cells. **e** Absolute numbers (±SEM) of splenic env-reactive donor EF4.1 CD4^+^ T cells over time after transfer into WT recipients and FV infection or B3-A^b^:env_123−139_ immunization (*n* = 3–4 mice per time point). **f** IL-7Rα and CD62L expression (*left*) and mean frequency (±SEM) of T_EM_ and T_CM_ populations in the same donor EF4.1 CD4^+^ T cells as in **e**. In **a**–**d**, each symbol represents an individual mouse

B3-A^b^:env_123–139_ cells were similarly efficient at in vivo priming of env_122–141_-reactive CD4^+^ T-cell clones from the semipolyclonal repertoire of TCRβ-transgenic EF4.1 mice,^32,36^ inducing their peak in vivo clonal expansion in WT adoptive hosts at levels equivalent to those induced by FV infection (Fig. 2a). Consistent with the in vitro data, B3-A^b^:env_123–139_ cells caused preferential expansion of high-affinity Vα2^+^ EF4.1T cell clonotypes, comparable with that following FV infection (Fig. 2b). Preferential in vivo expansion of high-affinity clonotypes in response to B3-A^b^:env_123–139_ vaccination was additionally confirmed with EVα2 and EVα3 CD4^+^ T cells, with only the former being efficiently primed (Fig. S5). The in vivo priming efficiency of B3-A^b^:env_123–139_ cells was independent of MHC II expression in the host, as also observed when mice lacking all conventional MHC II genes (*H2*^*dlAb1-Ea*^ mice)^37^ were used as hosts (Fig. 2c). Further arguing against indirect presentation of A^b^:env_123–139_ complexes by host APCs, B3-A^b^:env_123–139_ cells primed significantly higher peak expansion of EF4.1T cells than either B3-gp70 cells, which can only prime through gp70 processing and indirect presentation by host APCs, or B3-A^b^;gp70 cells, which can prime by gp70 processing and presentation by both B3 cells and host APCs (Fig. 2d).

Together, these findings suggest that the overexpression of A^b^:env_123–139_ complexes on nonprofessional APCs is sufficient for direct and effective priming of CD4^+^ T cells in vitro and in vivo. Furthermore, CD4^+^ T cells primed by B3-A^b^:env_123–139_ cells formed a memory pool, which was comparable in number with the chronic CD4^+^ T-cell response to FV infection (Fig. 2e) or other F-MLV gp70 vaccines^38,39^ and consisted of high frequencies of effector-memory (T_EM_) and central-memory (T_CM_) T cells, defined by patterns of CD62L and IL-7Rα (CD127) expression (Fig. 2f), thus validating efficient priming and memory formation in response to B3-A^b^:env_123–139_ vaccination.

### Amplified signal 1 sufficiently promotes polyfunctional Th1 cells

**Fig. 3:**
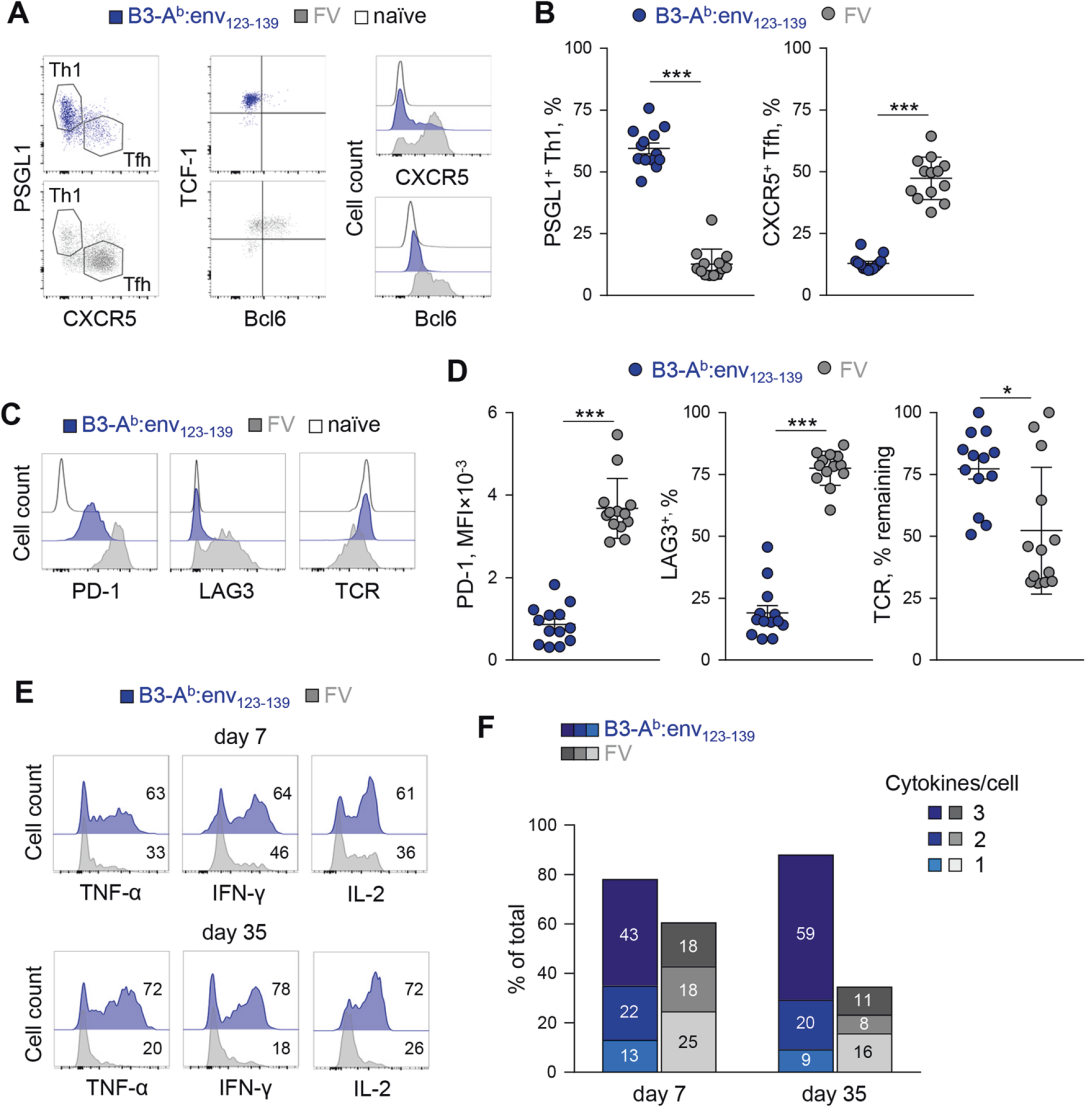
Polyfunctional Th1 cells preferentially develop in response to amplified signal 1. **a** Mutually exclusive expression of PSGL1 and CXCR5, defining Th1 and Tfh phenotypes, respectively, and expression of TCF-1 and Bcl6 in donor env-reactive EF4.1 CD4^+^ T cells 7 days after transfer into WT recipients and infection with FV or immunization with B3-A^b^:env_123−139_ cells. Host naïve CD4^+^ T cells are also included for comparison. **b** Frequency (±SEM) of Th1 (PSGL1^+^ CXCR5^−^) and Tfh (PSGL1^−^ CXCR5^+^) cells in the same donor EF4.1 CD4^+^ T cells as in **a**. **c** PD-1, LAG3 and TCR expression in the same donor EF4.1 CD4^+^ T cells as in **a**. **d** Mean fluorescence intensity (MFI) (±SEM) of PD-1 staining and frequency (±SEM) of cells expressing LAG3 or downregulated TCR in the same donor EF4.1 CD4^+^ T cells as in **a**. **e** Cytokine production following 4 h of in vitro restimulation with PdBu and ionomycin of donor env-reactive EF4.1 CD4^+^ T cells 7 and 35 days after transfer into WT recipients and FV infection or B3-A^b^:env_123−139_ immunization. **f** Frequency of cells producing 1, 2 or all 3 of the tested cytokines in the same donor EF4.1 CD4^+^ T cells as in **e**. In **b** and **d**, each symbol represents an individual mouse

Given the unexpected efficiency with which B3-A^b^:env_123–139_ cells primed clonal expansion and memory formation of env-reactive CD4^+^ T cells, we next examined their effect on CD4^+^ T-cell functional differentiation. Surprisingly, priming of EF4.1T cells treated with B3-A^b^:env_123–139_ cells exhibited a strong bias toward Th1 differentiation at the expense of Tfh differentiation, as judged by the expression of the subset-defining markers PSGL1, CXCR5 and Bcl6 (Fig. 3a, b; Fig. S6a, b). This was in stark contrast to the priming of EF4.1T cells during natural FV infection, which predominantly induces Tfh differentiation^38^ (Fig. 3a, b; Fig. S6a, b). Moreover, B3-A^b^:env_123–139_-primed CD4^+^ T cells maintained homogenously high levels of TCF-1 expression (Fig. 3a; Fig. S6a), arguing against terminal differentiation and consistent with subsequent memory formation (Fig. 2e, f). Despite their dominant Th1 profile, exemplified by high T-bet and IFN-γ expression, B3-A^b^:env_123–139_-primed CD4^+^ T cells did not express high levels of other markers associated with extreme or short-lived effector Th1 polarization, such as SLAM, CXCR6 or Ly6C, and did not acquire granzyme B expression, characteristic of cytotoxic function (Fig. S6a, b). In contrast, the latter markers were abundantly expressed in CD4^+^ T cells primed by FV infection in lymphopenic hosts^38^ (Fig. S6a, b).

Priming by B3-A^b^:env_123–139_ cells or FV infection resulted in further phenotypic and functional differences in effector EF4.1T cells. In contrast to FV-primed EF4.1T cells, which express high levels of the inhibitory receptors PD-1 and LAG3 and downregulate their TCR,^36,38^ B3-A^b^:env_123–139_-primed EF4.1T cells did not lose TCR expression or gain LAG3 expression and expressed modest amounts of PD-1 (Fig. 3c, d). The strong bias for Th1 differentiation and lack of inhibitory receptor expression following B3-A^b^:env_123–139_ vaccination was not restricted to EF4.1TCRβ-transgenic CD4^+^ T cells. Indeed, similar phenotypes were also induced by B3-A^b^:env_123–139_ cells in the fully polyclonal nontransgenic CD4^+^ T cells when they were monitored with the use of an A^b^:env_123–141_ tetramer (Fig. S7a, b).

Consistent with these patterns, priming by B3-A^b^:env_123–139_ cells conferred TNF-α, IFN-γ and IL-2 cytokine recall potential in a higher proportion of EF4.1T cells and at a higher per cell degree than priming by FV infection (Fig. 3e; Fig. S6a, b). More importantly, EF4.1T cells coexpressing all three cytokines were the largest subset following B3-A^b^:env_123–139_ immunization, but not FV priming, and this polyfunctionality was well preserved 35 days post priming with B3-A^b^:env_123–139_ cells, again in contrast to FV priming (Fig. 3e, f). These results indicated the increased availability of cognate pMHC presented by nonprofessional APCs, such as B3-A^b^:env_123–139_ cells and primed effector and memory Th1 CD4^+^ T cells that were superior to those primed by FV infection. To extend these findings to a different combination of TCR and pMHC II, we generated B3 cells presenting the A^b^:ova_323–339_ complex, an epitope from ovalbumin that is recognized by OT-II TCRαβ-transgenic T cells.^40^ B3-A^b^:ova_323–339_ cells stimulated OT-II CD4^+^ T cells in vitro and induced clonal expansion and a Th1 phenotype in vivo, similar to stimulation of EF4.1 CD4^+^ T cells by B3-A^b^:env_123–139_ cells (Fig. S8), indicating that this type of response to optimal signal 1 is a general property.

**Fig. 4:**
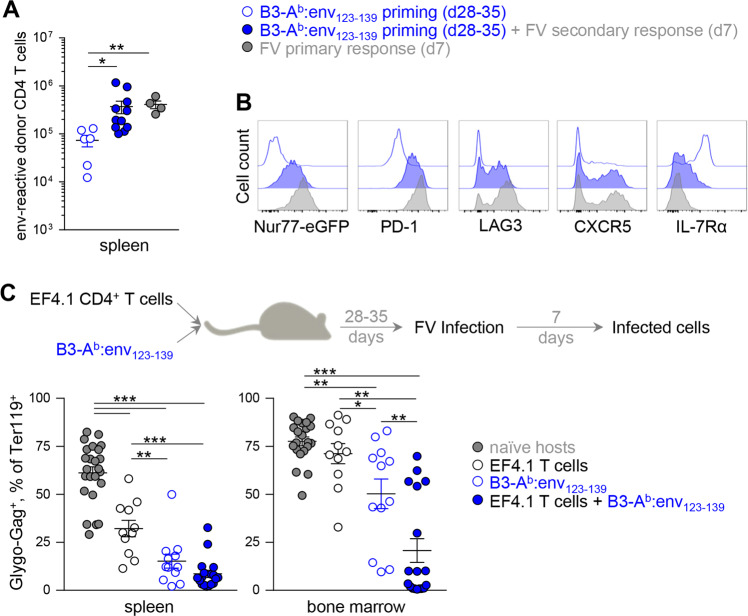
Protective anti-retroviral CD4^+^ T-cell responses induced by B3-A^b^:env_123−139_ immunization. **a** Absolute numbers (±SEM) of donor EF4.1 Nur77-eGFP doubly transgenic CD4^+^ T cells 28–35 days after transfer into WT recipients and B3-A^b^:env_123−139_ priming, with or without 7-day rechallenge with FV infection. Absolute numbers reached at the peak (day 7) of the primary response to FV infection are also shown for comparison. **b** Expression of PD-1, LAG3, CXCR5 and IL7Rα and of Nur77-reporting eGFP in the same donor EF4.1 CD4^+^ T cells as in **a**. **c** WT hosts were left untreated, received EF4.1 CD4^+^ T cells immunized with B3-A^b^:env_123−139_ cells or received EF4.1 CD4^+^ T cells and B3-A^b^:env_123−139_ immunization. At the memory phase (28–35 days later), mice were rechallenged with FV. Frequency (±SEM) of infected (Glyco-Gag^+^) cells in splenic (*left*) or bone marrow (*right*) Ter119^+^ cells is shown. In **a** and **c**, each symbol represents an individual mouse

To confirm the protective capacity of Th1 CD4^+^ T cells primed by B3-A^b^:env_123–139_ cells, we first examined their response to secondary challenge with FV. Compared with the numbers of memory EF4.1 T cells 4–5 weeks after B3-A^b^:env_123–139_ vaccination, acute FV infection-induced substantial clonal expansion to levels comparable with that of the primary response to FV (Fig. 4a). Secondary challenge of FV-infected mice with FV was not included for comparison since primary FV infection had not cleared at this time point.^33^ Moreover, memory EF4.1T cells that had been primed by B3-A^b^:env_123–139_ vaccination expressed activation markers similar to naïve EF4.1T cells after they both responded to acute FV infection (Fig. 4b), suggesting that these cells robustly and flexibly responded to secondary challenge. Following FV infection, B3-A^b^:env_123–139_-primed memory cells EF4.1T cells expressed lower levels of PD-1 and received overall reduced TCR signaling, as assessed by a Nur77-eGFP reporter transgene,^41^ than previously naïve EF4.1T cells (Fig. 4b), likely due to differences in FV loads between the two groups. Indeed, the presence of B3-A^b^:env_123–139_-primed memory cells EF4.1T cells had a strong protective effect against FV infection (Fig. 4c). Adoptive transfer of naïve EF4.1T cells alone reduced the viral loads in the spleen of recipient mice, as previously described,^42^ but not in the bone marrow, where a high proportion of infected cells were still found. By priming host CD4^+^ T cells, B3-A^b^:env_123–139_ vaccination alone induced significantly stronger protection in hosts that did not receive EF4.1T cells than transfer of naïve EF4.1T cells, both in the spleen and the bone marrow (Fig. 4c). Last, the combination of EF4.1T cells and B3-A^b^:env_123–139_ vaccination induced the strongest protection, with a near complete lack of infected cells detected by flow cytometry in either the spleen or the bone marrow in half of the recipient mice (Fig. 4c). Thus, priming of EF4.1T cells or nontransgenic host CD4^+^ T cells by B3-A^b^:env_123–139_ cells induced memory cells that were strongly protective against FV challenge.

### Full CD4^+^ T-cell priming by optimal signal 1 independent of prolonged presentation

**Fig. 5:**
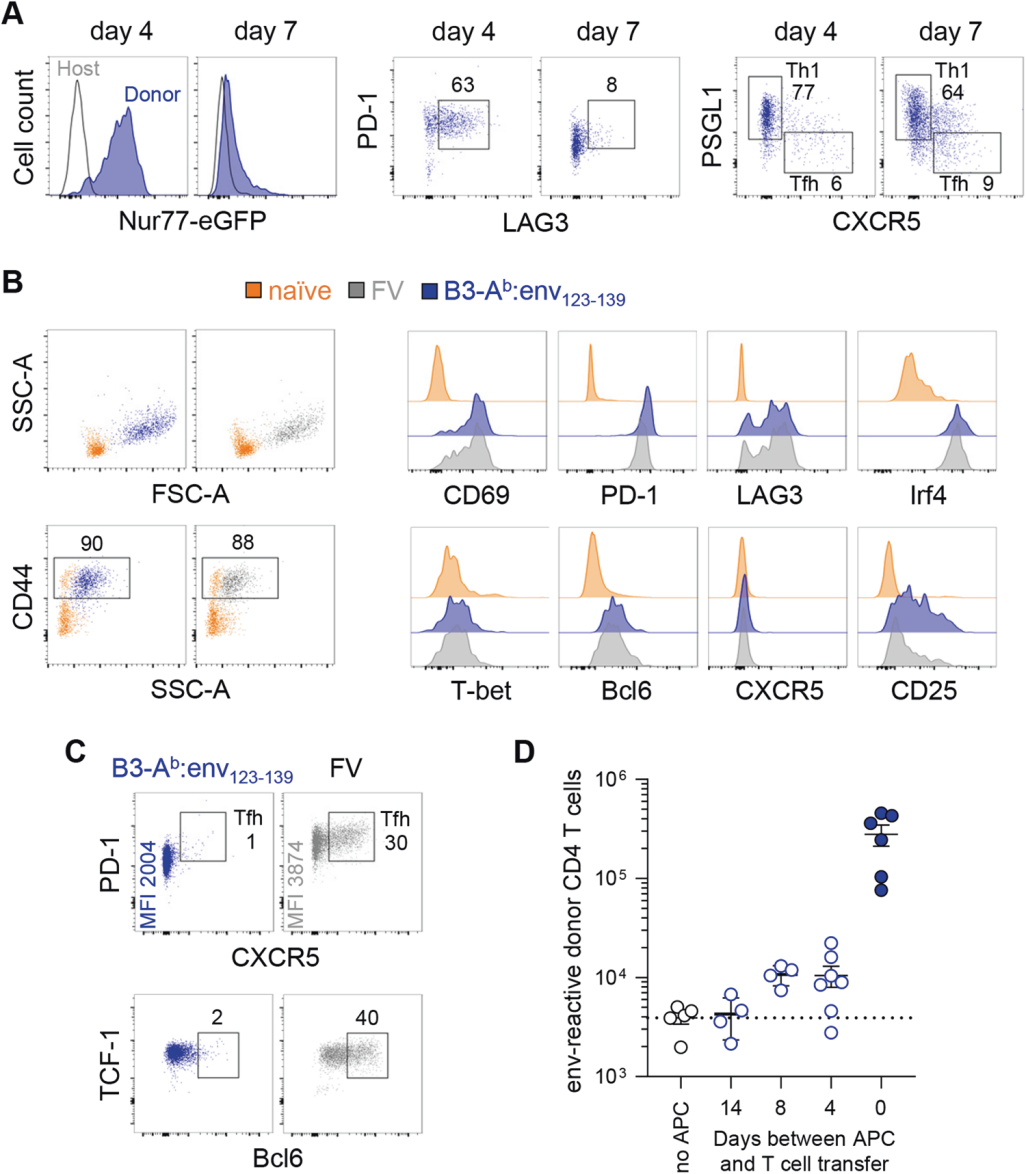
Efficient CD4^+^ T-cell priming by optimal signal 1 independent of prolonged presentation. **a** Expression of PD-1, LAG3, CXCR5 and PSGL1 and of Nur77-reporting eGFP in donor EF4.1 Nur77-eGFP doubly transgenic CD4^+^ T cells 4 and 7 days after transfer into WT recipients and B3-A^b^:env_123–139_ immunization. Nontransgenic host CD4^+^ T cells were used as a negative control for eGFP expression. **b** Expression of the indicated marker in donor monoclonal EVα2 CD4^+^ T cells 2 days after transfer into WT recipients and FV infection (*n* = 6) or B3-A^b^:env_123−139_ immunization (*n* = 6). Naïve host CD4^+^ T cells were included as controls. **c** Expression of PD-1, CXCR5, TCF-1 and Bcl6 in similar transfers of donor EVα2 CD4^+^ T cells as in **b** on day 4 after transfer and infection or immunization. **d** Absolute numbers (±SEM) of donor EF4.1 CD4^+^ T cells 7 days after transfer into WT hosts that had been immunized with B3-A^b^:env_123−139_ cells 0–14 days previously or into naïve WT hosts (no APC). Symbols represent individual mice pooled from two independent experiments

The efficiency with which B3-A^b^:env_123–139_ cells primed env-reactive CD4^+^ T cells was unexpected given their nonprofessional APC nature, but we reasoned that the lack of additional signals provided directly by B3-A^b^:env_123–139_ cells might have been compensated in vivo by increased strength and/or duration of signal 1. To dissect the underlying mechanism, we monitored the potency and duration of TCR signaling in EF4.1T cells responding to B3-A^b^:env_123–139_ cells. On day 4 post B3-A^b^:env_123–139_ vaccination, donor EF4.1T cells expressed homogeneously high levels of Nur77-driven eGFP (Fig. 5a), indicating that they had received strong TCR signals. However, Nur77-eGFP expression was no longer present on day 7 (Fig. 5a), suggesting that TCR signaling in donor EF4.1T cells had ceased prior to this time point. Similar results were obtained with staining for PD-1 and LAG3, which were highly expressed in responding EF4.1T cells on day 4 but not on day 7 after B3-A^b^:env_123–139_ vaccination, whereas the Th1 phenotype was maintained between days 4 and 7 (Fig. 5a).

Given the long protein half-life of eGFP (~1 day), it was possible that the high Nur77-eGFP expression on day 4 was indicative of TCR signaling that had already ceased as early as day 2 or 3 after priming, consistent with findings regarding Nur77-eGFP kinetics in other systems.^43^ The physiologically low frequency of env-reactive CD4^+^ T cells in EF4.1T cell transfers did not allow monitoring of the earliest time points before clonal expansion had occurred. We therefore switched to monoclonal EVα2 T cells, which we transferred at a higher frequency. We first confirmed that, similar to EF4.1T cells, EVα2 T cells assumed a Th1 phenotype and expressed low levels of PD-1 and LAG3 at the peak (day 7) of their response to B3-A^b^:env_123–139_ vaccination, in contrast to FV infection, which induced the opposite phenotypes (Fig. S9). EVα2 T cells became fully and comparably activated 2 days after priming with either B3-A^b^:env_123–139_ cells or FV infection (Fig. 5b). Among typical activation markers, the high expression of PD-1 and LAG3, indicators of TCR signal strength, as well as of Irf4, an indicator of accumulated TCR signaling, was notable.^43,44^ By day 4, B3-A^b^:env_123–139_-primed EVα2 T cells had downregulated PD-1 and no longer expressed LAG3 or Bcl6, in contrast to FV-primed EVα2 T cells, which maintained high levels of PD-1 and Bcl6 expression and acquired CXCR5 expression, indicative of Tfh differentiation (Fig. 5c). Together, these results suggested that B3-A^b^:env_123–139_ vaccination delivered strong TCR signaling, but for a short duration, not exceeding the first 4 days.

To examine whether the apparent short duration of antigen presentation by B3-A^b^:env_123–139_ cells was due to their elimination from adoptive hosts, we delayed the transfer of env-reactive donor CD4^+^ T cells relative to B3-A^b^:env_123–139_ transfer. Clonal expansion of EF4.1T cells was already severely compromised if the cells were transferred 4 days after B3-A^b^:env_123–139_ transfer (Fig. 5d), indicating that A^b^:env_123–139_ was no longer present at this time point. Thus, B3-A^b^:env_123–139_ vaccination induced a lasting and protective Th1 memory population, despite the short duration of antigen presentation.

### Polyfunctional Th1 cells elicited by enhanced signal 1 on diverse APCs

We attributed the priming ability of B3-A^b^:env_123–139_ cells to the enhanced properties of the presented pMHCs, but it was important to evaluate the contribution of B3 cells, which might not be typical APCs. To this end, we used another pro-B cell leukemia cell line, F6,^35^ also lacking expression of MHC II and costimulatory molecules (Fig. S10a, b), and found that F6-A^b^:env_123–139_-transduced cells stimulated EVα2 T cells in vitro as efficiently as B3-A^b^:env_123–139_ cells (Fig. S10b, c). We also used hematopoietic stem and progenitor cells (HSPCs) from the bone marrow of *H2*^*dlAb1-Ea*^ mice lacking endogenous MHC II. Upon HSPC transduction and further in vitro differentiation, we isolated two distinct populations, namely, CD11c^+^ DCs and CD11c^−^ non-DCs, expressing intermediate and high levels of A^b^:env_123–139_, respectively (Fig. S11a, b). As expected, BM-DCs expressed substantially higher levels of the accessory molecules CD80, CD86, OX40L, ICOS-L and PD-L1 than BM-non-DCs (Fig. S11c).

**Fig. 6:**
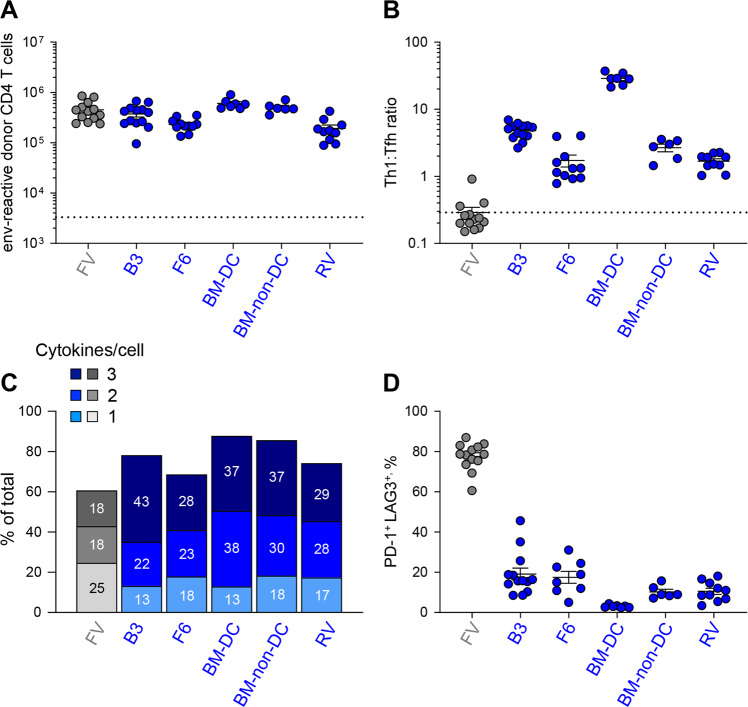
Polyfunctional Th1 cells elicited by enhanced signal 1 on diverse APCs. EF4.1 CD4^+^ T cells were adoptively transferred into WT recipients infected with FV (*n* = 13) or immunized with B3 cells (*n* = 13), F6 cells (*n* = 11), *H2*^*dlAb1-Ea*^ BM-DCs (*n* = 7) or *H2*^*dlAb1-Ea*^ BM-non-DCs (*n* = 6), all transduced with an A^b^:env_123−139_-expressing vector or WT recipients injected directly with retroviral particles encapsulating the A^b^:env_123−139_-expressing vector (RV) (*n* = 10). **a** Absolute numbers (±SEM) of donor EF4.1 CD4^+^ T cells 7 days after transfer. **b** Tfh:Th1 ratio (determined by expression of PSGL1 and CXCR5) in donor EF4.1 CD4^+^ T cells 7 days after transfer. **c** Frequency of cells producing 1, 2 or all 3 of the tested cytokines (TNF-α, IFN-γ and IL-2) in the in vitro restimulated donor EF4.1 CD4^+^ T cells 7 days after transfer. **d** Frequency (±SEM) of PD-1^+^ LAG3^+^ cells in donor EF4.1 CD4^+^ T cells 7 days after transfer. the data shown were pooled from three to four independent experiments

When transduced with the A^b^:env_123–139_-expressing vector and used as vaccines, F6 cells, as well as BM-DCs and BM-non-DCs, induced robust clonal expansion of EF4.1T cells, similar to that induced by B3-A^b^:env_123–139_ cells or by FV infection (Fig. 6a). Notably, direct intravenous injection of the A^b^:env_123–139_-expressing retroviral vector into recipient mice also induced EF4.1T cell clonal expansion (Fig. 6a), indicating that A^b^:env_123–139_ expression was sufficient for priming of env-reactive T cells, irrespective of APC type. Importantly, in contrast to the strong Tfh bias following FV infection, A^b^:env_123–139_ presentation by all APCs tested promoted predominantly Th1 differentiation (Fig. 6b) and significantly high frequencies of CD4^+^ T cells with TNF-α, IFN-γ and IL-2 cytokine recall potential (Fig. 6c), without inducing expression of PD-1 and LAG3 (Fig. 6d).

While A^b^:env_123–139_-expressing BM-DCs were superior to other A^b^:env_123–139_-expressing APCs based on all the read-outs employed here, it was noteworthy that preferential priming of polyfunctional Th1 cells seemed to be an intrinsic property of the A^b^:env_123–139_-expressing vector, rather than the APC type. Indeed, the CD4^+^ T-cell response induced by this vector (expressed by any APC type or administered directly) comprised between 40% and 90% (median 60%) Th1 phenotype cells. This was in contrast not only to FV infection but also to a great variety of other immunization regimens. Indeed, our previous work^38,45^ demonstrated that Th1 cells typically comprise a median of 22% (between 10% and 30%) of the CD4^+^ T-cell response to other immunization regimens, including a human adenovirus 5 (Ad5)-based vaccine vector and a replication-competent mouse cytomegalovirus (mCMV)-based vector (both expressing F-MLV gp70), FV-induced FBL-3 leukemia cells expressing F-MLV gp70, and env_122–141_ peptide immunization in the Sigma Adjuvant System.

### Amplified signal 1 expands otherwise unresponsive tumor-reactive CD4^+^ T cells

As Th1 cells are linked with good prognosis in all cancer types,^17^ we next investigated whether enhanced signal 1 would promote a protective Th1 response when presented by cancer cells in general or whether cancer cells can subvert such Th1-promoting signals. To test the immunogenicity of presentation by cancer cells other than the B3 and F6 pro-B cell leukemias, we used B16 melanoma cells. The expression of A^b^:env_123–139_ in B16 cells had no measurable effect on their growth after transplantation into WT mice (Fig. S12a). Similar results were also obtained when A^b^:env_123–139_ was expressed in MCA-38 colon adenocarcinoma cells (Fig. S12b), indicating either lack of env-reactive CD4^+^ T-cell priming or ineffectiveness of primed T cells to reject these solid tumors.

**Fig. 7:**
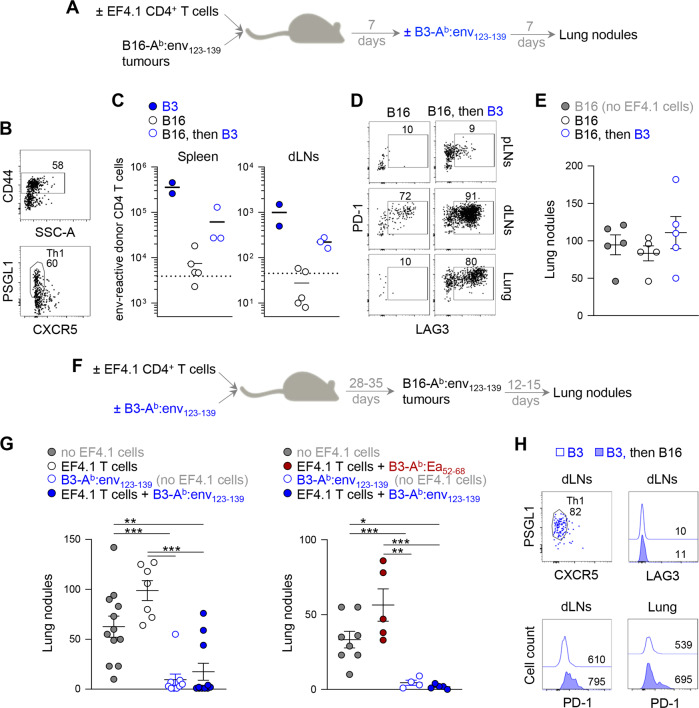
Antitumoral activity of CD4^+^ T cells primed by enhanced signal 1 on tumor or B3 cells. **a** WT hosts were injected intravenously with B16-A^b^:env_123–139_ melanoma cells, with or without concurrent transfer of EF4.1 CD4^+^ T cells. Some of these hosts received B3-A^b^:env_123–139_ immunization 7 days after B16 melanoma cell challenge, and all mice were examined for the presence of lung nodules 14 days after melanoma cell challenge. **b** CD44, PSGL1 and CXCR5 expression in donor EF4.1 CD4^+^ T cells isolated from the spleens of B16-A^b^:env_123–139_ tumor-bearing WT hosts. **c** Absolute numbers of donor EF4.1 CD4^+^ T cells in the spleens or lung-draining lymph nodes (dLNs) of WT host mice that received only B3-A^b^:env_123–139_ immunization (B3), only B16-A^b^:env_123–139_ melanoma challenge (B16) or B16-A^b^:env_123–139_ melanoma challenge followed by B3-A^b^:env_123–139_ immunization (B16 then B3). **d** PD-1 and LAG3 expression in the same donor EF4.1 CD4^+^ T cells as in **c**. Cells isolated from peripheral lymph nodes (pLNs) are also shown for comparison. **e** Number of lung nodules (±SEM) in WT mice challenged with B16-A^b^:env_123–139_ melanoma cells only (B16 (no EF4.1 cells)) or in those that received EF4.1 CD4^+^ T cells and either B16-A^b^:env_123−139_ melanoma challenge (B16) or B16-A^b^:env_123–139_ melanoma challenge followed by B3-A^b^:env_123–139_ immunization (B16 then B3). **f** WT hosts were left untreated or received EF4.1 CD4^+^ T cells with or without concurrent immunization with B3-A^b^:env_123–139_ or control B3-A^b^:Ea_52–68_ cells. All mice were challenged with B16-A^b^:env_123–139_ melanoma cells 28–35 days later and examined for the presence of lung tumors 12–15 days after melanoma challenge. **g** Number of lung nodules (±SEM) in the indicated combination of EF4.1 CD4^+^ T-cell transfer and immunization. **h** PSGL1, CXCR5 and LAG3 expression in donor EF4.1 CD4^+^ T cells from dLNs and PD-1 expression in donor EF4.1 CD4^+^ T cell dLNs and lungs of WT recipients that were only immunized with B3-A^b^:env_123–139_ cells (B3) or were additionally challenged with B16-A^b^:env_123–139_ melanoma cells (B3 then B16)

These findings are in agreement with prior reports using TCRαβ-transgenic CD4^+^ T cells reactive with the melanocyte antigen TRP-1.^46,47^ The failure of naïve TRP-1-specific CD4^+^ T cells to reject B16 cells in WT hosts has been interpreted as an inability of B16 cells to prime naïve CD4^+^ T cells without tumor antigen presentation by host APCs.^48^ However, when tested in vitro, B16-A^b^:env_123–139_ cells induced CD69 expression in EVα2 T cells reasonably efficiently (Fig. S13), suggesting that they could prime naïve CD4^+^ T cells under these conditions. Similarly, intravenous B16-A^b^:env_123–139_ challenge of host mice activated concurrent transfer of EF4.1T cells (assessed by upregulation of CD44 expression), which also displayed a Th1 phenotype (Fig. 7a, b). However, despite their activated phenotype, the accumulation of EF4.1T cells was very limited, particularly in the lymph nodes draining the lungs, where B16 cells had established tumors and where EF4.1 cells also expressed high levels of PD-1 and LAG3 (Fig. 7c, d). Accordingly, transfer of EF4.1T cells did not protect against B16-A^b^:env_123–139_ challenge (Fig. 7e), suggesting that priming under these conditions was either ineffective or tolerant. We therefore examined whether provision of enhanced signal 1 by highly immunogenic B3-A^b^:env_123–139_ vaccination could boost the numbers of env-reactive CD4^+^ T cells that had been ineffectively primed or tolerized by B16-A^b^:env_123–139_ tumors. Injection of B3-A^b^:env_123–139_ cells 7 days after B16-A^b^:env_123–139_ challenge led to substantial expansion of EF4.1T cells in the spleen and lung-draining lymph nodes, albeit not reaching the levels seen by B3-A^b^:env_123–139_ vaccination of tumor-free mice, and to accumulation in the lungs (Fig. 7c, d). The expanded EF4.1T cells continued to express high levels of PD-1 and LAG3 and failed to protect against B16-A^b^:env_123–139_ growth (Fig. 7d, e), demonstrating that this failure was not due to lack of EF4.1T cell priming.

It remained possible that EF4.1T cells were unsuitably differentiated following initial priming by B16-A^b^:env_123–139_ cells or that they failed to effectively recognize the cells even after boosting with B3-A^b^:env_123–139_ cells. To distinguish between these two possibilities, we established a functional Th1 memory pool by B3-A^b^:env_123–139_ vaccination of recipient mice 4–5 weeks prior to B16-A^b^:env_123–139_ challenge (Fig. 7f). Transfer of EF4.1T cells alone in these hosts did not provide any protection against B16-A^b^:env_123–139_ challenge and was even associated with an increase in lung tumors (Fig. 7g). In stark contrast, vaccination with B3-A^b^:env_123–139_ cells rendered the host mice almost completely resistant to B16-A^b^:env_123–139_ challenge, and this protection was observed even in hosts that had not received EF4.1T cells (Fig. 7g). The protective effect of B3-A^b^:env_123–139_ vaccination was dependent on A^b^:env_123–139_ expression and was not mediated by cross-reactive tumor antigens shared between B3 and B16 cells, most notably, endogenous retroviruses,^49^ as it was not observed following vaccination with B3-A^b^:Ea_52–68_ cells (Fig. 7g). Importantly, the protective capacity of B3-A^b^:env_123–139_-primed EF4.1T cells was accompanied by retention of the Th1 phenotype, lack of LAG3 expression and only modest induction of PD-1 expression in draining lymph nodes or the lungs following B16-A^b^:env_123–139_ challenge (Fig. 7h). Together, these results emphasize the importance of appropriate Th differentiation in the protection against solid tumors and the ability of amplified signal 1 to immunize against tumor antigens that would not be otherwise targeted effectively.

## Discussion

CD4^+^ T cells integrate multiple signals, which collectively determine clonal expansion and helper subset differentiation.^3,4^ Signal 1 confers antigen specificity but may also be the rate-limiting factor in the initiation of CD4^+^ T-cell responses, owing to the rarity of cognate pMHC. Our findings suggest that ample presentation of an optimal pMHC II complex is sufficient to induce an effector and memory CD4^+^ T-cell response in the absence of classic costimulation by the same APC. More importantly, they further demonstrate that enhanced signal 1 alone preferentially promotes polyfunctional Th1 cells and significantly improves protective immunity compared with standard vaccines or immunization regimens.

These findings have a number of theoretical and practical implications. First, they seem at odds with the accepted notion that presentation by nonprofessional APCs, lacking accessory signals, is tolerogenic rather than immunogenic.^3,4^ Particularly in the case of pre-B cell leukemia, the absence of costimulatory molecule expression has been shown to induce anergy rather than activation of primed T cells.^50^ However, subsequent studies incriminated indirect presentation of B cell lymphoma antigens by host APCs in the induction of CD4^+^ T-cell tolerance, whereas direct presentation by B cell lymphoma cells led to efficient CD4^+^ T-cell priming.^51,52^ Moreover, injection of nonprofessional APCs, in the form of fibroblasts, has long been demonstrated to prime CD8^+^ T cells without the involvement of host APCs, provided they reach lymphoid organs where costimulation and cytokine signals can be delivered separately from signal 1.^53^ Therefore, costimulation and cytokine signals provided by APCs that do not present antigens suffice to allow T-cell priming by the provision only of ample signal 1 on separate APCs.

Our results with B3 and F6 pro-B cell leukemia cells are entirely consistent with these prior reports that direct presentation by B cell lymphoma cells is immunogenic.^51,52^ The expression of covalently linked pMHC complexes in B3 and F6 precludes indirect presentation of the source antigen gp70, which is not present in this system. Moreover, host expression of MHC II was not required for CD4^+^ T-cell priming by B3-A^b^:env_123–139_ cells, and indirect presentation of gp70 expressed by MHC II-negative B3 cells did not induce a comparable CD4^+^ T-cell response. While indirect presentation is unlikely to contribute in this setting, preformed pMHC complexes can also be transferred between cells, a phenomenon known as cross-dressing, and contribute to CD4^+^ T-cell priming by tumor cell vaccines.^54^ Transfer of pMHC complexes to host DCs cannot be ruled out, but several observations argue against a critical requirement. B3-A^b^:env_123–139_ cells activated primary CD4^+^ T cells in vitro in the absence of other APCs. Antigen presentation following B3-A^b^:env_123–139_ cell inoculation ceased as quickly as the cells disappeared and was not continued by pMHC complexes transferred to long-lived APCs. DCs engineered to express A^b^:env_123–139_ complexes induced a stronger and more Th1 polarized response than B3-A^b^:env_123–139_ cells. Last, efficient CD4^+^ T-cell priming was observed with B3 and F6 pro-B cell leukemia cells but not with B16 melanoma or MCA-38 colon adenocarcinoma cells, although all these tumor cell lines can cross-dress DCs.^54^

These differences between cell types indicate that A^b^:env_123–139_ complexes are presented primarily by the cells that express them. Nevertheless, with the possible exception of presentation by B16 and MCA-38 cells, these complexes appeared far more immunogenic than all other vaccines and immunization regimens we have previously used in the FV infection model, including viral-vectored, peptide-based and cell-based vaccines.^38,45^ In this context, the type of cell presenting the pMHC complexes or the means by which it acquired them are less important than the overall amount of pMHC presented. Consistent with this concept, direct injection of a noninflammatory,^55^ replication-defective retroviral vector encoding A^b^:env_123–139_ complexes was sufficient to prime env-specific CD4^+^ T cells.

Although priming by provision of optimal signal 1 alone induced the clonal expansion, polyfunctional Th1 phenotype and protective capacity of the responding CD4^+^ T cells, it is important to note that the degree of stepwise differentiation of the Th1 phenotype appeared intermediate or incomplete. Despite T-bet and IFN-γ expression, which typify Th1 differentiation, CD4^+^ T cells primed by optimal signal 1 alone retained full expression of TCF-1 and increased expression of CD127 (IL-7Rα) and CD62L and lacked expression of markers associated with more advanced or terminal Th1 differentiation, such as Ly6C and Granzyme B or the inhibitory receptors PD-1 and LAG3. These data suggest that the provision of ample signal 1 induces intermediate Th1 differentiation, with a memory rather than short-lived effector CD4^+^ T-cell phenotype, which might underlie the strong recall responses observed. Bypassing the short-lived effector stage in immunization with signal 1 alone might also be beneficial in reducing any adverse effects of inflammatory CD4^+^ T-cell responses to the immunization itself while still achieving clonal expansion and appropriate polarization of protective CD4^+^ T cells. The partial activation and Th1 differentiation of CD4^+^ T cells by provision of signal 1 alone is also consistent with preferential expansion of high-affinity clonotypes. Such clonotypic bias might be beneficial in cases where protective capacity is correlated with TCR affinity but detrimental when TCR repertoire diversity is a requirement for the response to quickly evolving viruses or tumors.

A notable exception in effective CD4^+^ T-cell priming was the presentation of A^b^:env_123–139_ complexes by B16 and MCA-38 cells. The defect appeared to be at the level of effector CD4^+^ T-cell accumulation during initial in vivo priming. Indeed, naïve env-reactive CD4^+^ T cells were fully activated in vitro and exhibited the hallmarks of antigen experience in vivo when primed by B16-A^b^:env_123–139_ cells but failed to accumulate in high numbers and displayed an exhausted phenotype. Moreover, memory env-reactive CD4^+^ T cells, generated by B3-A^b^:env_123–139_ immunization, were highly effective in rejecting B16-A^b^:env_123–139_ melanoma cells, indicating that A^b^:env_123–139_ complexes expressed in B16 cells can be recognized effectively. These findings suggest that B16 and MCA-38 cells induce unsuitable and ineffective differentiation of responding CD4^+^ T cells. The defect in effector CD4^+^ T-cell expansion could be restored by B3-A^b^:env_123–139_ immunization of mice bearing established lung metastases, although this alone did not translate to tumor regression or control, and the expanded CD4^+^ T cells continued to express high levels of PD-1 and LAG3. The diminished protective effect against established lung metastases might relate to the nature of such solid tumors, which deploy several immune-suppressive mechanisms.^25,56^ Alternatively, it is likely that protective immunity against B16 and MCA-38 cells is compromised by the nonphysiological production of recombinant infectious MLVs by both these cell lines, which infect tumor-infiltrating T cells, as recently demonstrated.^49^ Nevertheless, the antigen-specific expansion of CD4^+^ T cells by B3-A^b^:env_123–139_ immunization even in tumor-bearing mice should enhance the therapeutic effect of very low doses of anti-PD-1 or anti-LAG3 immunotherapy, thereby avoiding immune-related adverse events arising from the nonspecific T-cell activation associated with high doses for such immunotherapy.^26^

T-cell immunity to tumors has traditionally been considered a function of CD8^+^ cytotoxic T cells, although CD4^+^ T cells can be equally efficient, if not more efficient, than CD8^+^ T cells in certain settings.^57^ Recent findings suggest a more important contribution of CD4^+^ T cells in antitumor immunity than previously appreciated.^58,59^ CD4^+^ T cells can develop cytotoxic activity, which acts synergistically with IFN-γ production to mediate antitumor immunity.^60^ F-MLV env-reactive CD4^+^ T cells protect against FV-induced erythroleukemia at least in part through IFN-γ effects on target cells^42^ and develop granzyme-mediated cytotoxic activity, particularly in lymphopenia or reduced regulatory T-cell activity.^45,61^ However, such cytotoxic activity is also associated with terminal or divergent Th1 differentiation^45^ and dependent on cytokines rather than TCR signaling.^60^ Consequently, CD4^+^ T cells primed by B3 cells presenting only optimal signal 1 did not acquire Granzyme B expression, which would indicate cytotoxic activity, and their antitumor activity is therefore likely mediated by IFN-γ, which they produced in very high amounts.

Our findings also highlight similarities between CD4^+^ and CD8^+^ T cells in terms of priming requirements and outcome, previously viewed as differences. CD8^+^ T cells recognize peptide epitopes of restricted length (8–12 amino acids) that can be presented by most cell types in the body, require relatively short stimulation periods to commit to programmed clonal expansion and are limited in their choices of functional differentiation, which is overwhelmingly type 1. In contrast, CD4^+^ T cells recognize peptide epitopes of highly variable length, ranging between 7 and 35 amino acids,^27–29^ and may even react with peptides shorter than 5 amino acids.^62^ Importantly, the presentation of very short peptides, as well as PFRs, may affect both the clonal composition and functional differentiation of responding CD4^+^ T cells. CD4^+^ T-cell antigen recognition is restricted to APC types expressing MHC II, which are considerably less diverse than cell types expressing MHC I. They are also thought to require a longer duration of continuous antigenic stimulation for full functional differentiation,^63–67^ although this requirement might be more pronounced for Tfh differentiation.^68^ Last, CD4^+^ T-cell functional differentiation is highly diverse, typically involving a balance of Th subsets.^5–9^ Presented with an optimal and abundant pMHC signal, CD4^+^ T cells behave similarly to CD8^+^ T cells. Full CD4^+^ T-cell clonal expansion and differentiation is induced by pMHC displayed on a variety of cell types, including nonprofessional APCs, for a short duration. Indeed, our results indicate that antigen presentation by B3-A^b^:env_123–139_ cells ends in the first 4 days. Longer presentation may well be compatible with CD4^+^ T-cell differentiation, but it is not necessary. Most notably, the CD4^+^ T-cell response to enhanced signal 1 was phenotypically monomorphic and strongly Th1 biased, similar to that of CD8^+^ T cells. Biased Th1 effector differentiation was observed despite strong TCR signaling, previously thought to promote Tfh differentiation or impede memory formation.^11–14^ Furthermore, Th1 effector cells induced by A^b^:env_123–139_ complexes developed into polyfunctional memory Th1 cells, which were highly protective against retroviral infection and tumor challenge. Indeed, a single B3-A^b^:env_123–139_ vaccination protected WT hosts against B16 challenge as effectively as repeated vaccinia virus-vectored immunization with the melanocyte antigen TRP-1^69^ or predifferentiated TRP-1-specific TCR-transgenic CD4^+^ T cells, in conjunction with anti-CTLA-4 treatment of lymphopenic hosts.^46,47^ Similarly, B3-A^b^:env_123–139_ vaccination achieved highly efficient protection against FV infection to a degree that was previously seen only in env-specific TCR-transgenic hosts.^42^

Together, our findings demonstrate the dominant effect of signal 1 in the induction of a protective Th1 response and argue that although the generation of cognate pMHC complexes is inherently limited in natural infection or cancer, it need not be limited in vaccination. Instead, based on these findings, we propose that increasing the abundance of signal 1 should be the primary aim of T-cell-based immunotherapies.

## Materials and methods

### Mice

Inbred C57BL/6J (B6) and CD45.1^+^ congenic B6 (B6. SJL-*Ptprca Pep3b*/BoyJ) mice were originally obtained from The Jackson Laboratory. CD45.1^+^ CD45.2^+^ WT B6 mice were obtained by intercrossing B6 and CD45.1^+^ congenic B6 mice. TCRβ-transgenic EF4.1 mice,^32^ TCRαβ-transgenic OT-II mice,^40^ Nur77-eGFP transgenic mice,^41^ TCRαβ-transgenic EVα2 and EVα3 mice,^33^ *Rag2*-deficient mice^70^ and mice constitutively lacking all conventional MHC II genes (*H2*^*dlAb1-Ea*^)^37^ were all in the B6 genetic background. Male or female mice, between 8 and 12 weeks of age, were used in separate sex-matched experiments. All mouse strains were maintained at the Francis Crick Institute’s animal facility. All animal experiments were approved by the ethical committee of the Francis Crick Institute and conducted according to local guidelines and UK Home Office regulations under the Animals (Scientific Procedures) Act 1986 (ASPA).

### Plasmids, transfections and retroviral transductions

pRV plasmids containing the cDNA sequences of the F-MLV envelope protein subunit gp70 (gp70) of the mouse *H2-Aa and H2-Ab1* chains alone (A^b^) or covalently linked with the F-MLV-env_123–139_ (EPLTSLTPRCNTAWNRL, A^b^:env_123–139_) or the Ea_52–68_ (ASFEAQGALANIAVDKA, A^b^:env_52–68_) epitopes were synthesized and sequenced by GENEWIZ (South Plainfield, NJ, USA). Transfection of Platinum-E retroviral packaging cells and retroviral transduction of target cells were performed as previously described.^71^

### Cell lines

Murine pro-B cell leukemia cell lines B3 and F6 were originally established from transgenic mice overexpressing IL-7 under the control of the MHC II (E alpha) promoter.^35^ These cells do not express endogenous MHC II or the MHC II promoter-driven IL-7 transgene. Both B3 and F6 cells induce full-strength leukemia upon transplantation into T cell- and B cell-deficient *Rag1*^−/−^ mice^71^ but are immunologically rejected in immunocompetent mice. Melanoma B6-derived B16-F0 cells (CRL-6322) were obtained from ATCC. MCA-38 cells were kindly provided by Dr Giorgio Trinchieri (National Cancer Institute, Bethesda, MD, USA). The F-MLV-env_122–141_ reactive T-cell hybridoma H5 was generated and characterized previously.^34^ Unless otherwise stated, all primary cell lines were cultured in complete Iscove’s modified Dulbecco’s medium (IMDM; 2 mM L-glutamine, 100 U penicillin, 0.1 mg/mL streptomycin, 10^−5^ M 2-mercaptoethanol (all from Sigma-Aldrich, St. Louis, MO, USA)) supplemented with 5% heat-inactivated fetal bovine serum (FBS) (Gibco by Thermo Fisher Scientific, Waltham, MA, USA).

### In vitro differentiation of hematopoietic stem and progenitor cells (HSPCs)

Bone marrow cell suspensions were cultured in complete IMDM supplemented with 20 ng/mL GM-CSF (PeproTech, Rocky Hill, NJ, USA). On days 7–9, loosely adhered and nonadherent cells, comprising 60–70% CD11c^+^ MHCII^+^ BMDCs, were harvested and used as APCs for CD4^+^ T-cell activation in coculture experiments. For retroviral transduction, HSPCs were enriched from *H2*^*dlAb1-Ea*^ bone marrow cell suspensions by immunomagnetic negative selection using the EasySep^TM^ Mouse Hematopoietic Progenitor Cell Isolation Kit (STEMCELL Technologies, Vancouver, Canada). After 4 h of resting in complete RPMI-1640 (Gibco) medium supplemented with 10% FBS and IL-3, IL-6 and SCF (from PeproTech, all at 50 ng/ml), isolated HSPCs were retrovirally transduced with the mouse *H2-Aa* and *H2-Ab1* chains covalently linked with the F-MLV-env_123–139_ peptide, as previously described.^71^ 48 h after transduction, HSPCs were seeded into DC differentiation medium (IMDM media supplemented with 5% FBS, 20 ng/mL GM-CSF, 100 ng/mL FLT3L and 10 ng/mL SCF). Transduction efficiency was assessed by the expression of MHC II and differentiation in the DC lineage by the expression of CD11b, CD11c and MHC II. Between days 5 and 7 of differentiation, CD11c^+^ MHC II^int^ (BM-DC) and CD11c^−^ MHC II^high^ (BM-non-DC) cells were purified by cell sorting in a BD FACSAria^TM^ Fusion instrument (BD Biosciences, Franklin Lakes, NJ, USA) before in vivo adoptive transfer.

### Primary CD4^+^ T-cell isolation and in vitro activation

Single-cell suspensions were prepared from the spleens and/or lymph nodes of EF4.1, EVα2 or EVα3 TCR transgenic mice, and CD4^+^ T cells were enriched using the immunomagnetic EasySep^TM^ PE Positive Selection Kit or Mouse CD4^+^ Positive Selection Kit (STEMCELL Technologies), with 90–95% purity. For in vitro activation, 1 × 10^5^ purified EVα2 or EVα3 CD4^+^ T cells were cocultured with 0.25 × 10^5^ APCs displaying A^b^:env_123–139_ complexes or with 0.25 × 10^5^ BMDCs preloaded with the env_122–141_ peptide (DEPLTSLTPRCNTAWNRLKL, 10 μM, 60–90 min at 37 °C). The hybridoma cell line H5 was stimulated under the same conditions. Alternatively, 2.5 × 10^6^ total splenocytes from EVα2 mice were stimulated with the indicated amounts of variable-length peptides spanning the core F-MLV env epitope. Where indicated, to assess lymphocyte proliferation, purified CD4^+^ T cells were labeled with CFSE (1–2.5 μM, Thermo Fisher Scientific), and APCs were irradiated (20 Gy) before coculture. Early T-cell activation and proliferation were assessed 18 h later by CD69 expression and 72 h later by CFSE dilution, respectively.

### Adoptive CD4^+^ T-cell transfer, infection, immunization and tumor challenge

Purified CD4^+^ T cells from TCR β-transgenic EF4.1 or Nur77-eGFP EF4.1 mice (1  × 10^6^ per recipient) or from TCRαβ-transgenic EVα2 and EVα3 mice (5 × 10^5^ or 2 × 10^5^ cells per host, for experiments where the phenotype was recorded respectively at day 2 or at day ≥ 4, respectively) were injected via the tail vein into CD45.1^+^ CD45.2^+^ B6 recipient mice. Transduced B3 and F6 cell lines (both 1.5 × 10^6^ per recipient) and BM-derived primary cells (2 × 10^5^) were adoptively transferred by intravenous injection. Stocks of FV, a retroviral complex of a replication-competent B-tropic F-MLV (F-MLV-B) and a replication-defective spleen focus-forming virus (SFFV), were prepared as previously described.^33^ For injection of A^b^:env_123–139_-transducing retroviral particles, 100 μL of Plat-E culture supernatants were intravenously injected into recipient mice. Unless otherwise specified, in vivo adoptive transfer of APCs and/or infection and immunization were performed the same day or a day apart from CD4^+^ T-cell adoptive transfer. Tumor challenge was initiated by subcutaneous inoculation of 5 × 10^5^ B16 or MCA-38 tumor cell suspensions into the right flank of CD45.1^+^ CD45.2^+^ WT mice. For the experimental model of lung metastasis, 3 × 10^5^ B16 cells were injected into the tail vein of CD45.1^+^ CD45.2^+^ WT mice. For counting of lung metastasis, lungs were harvested and fixed in Bouin’s solution; visible nodules were enumerated by a blinded observer.

### Flow cytometry

Single-cell suspensions were prepared from spleens and LNs (peripheral LNs: axillary, brachial and inguinal; lung-draining LNs: mediastinal) by mechanical digestion and from lung tissues by enzymatic digestion in 20 μg/mL Liberase (Sigma-Aldrich) and 50 μg/mL DNase I (STEMCELL Technologies) for 30 min at 37 °C followed by homogenization with a GentleMax dissociator (Miltenyi Biotec, Bergisch Gladbach, Germany). For the detection of intracellular cytokines, spleen cell suspensions were stimulated for 4 h with phorbol 14,13-dibutyrate (PdBu) and ionomycin (both at 0.5 μg/mL, from Sigma-Aldrich) in the presence of 5 μg/mL brefeldin A and 2 nM monensin (both from BioLegend, San Diego, CA, USA). Detection of Glygo-Gag^+^ FV-infected cells, CXCR5, surface staining and intracellular staining was performed as previously described.^38^ The F-MLV-env_123–141_ (EPLTSLTPRCNTAWNRLKL) A^b^-specific BV421-labeled tetramer was provided by the NIH Tetramer Core Facility. Tetramer staining was performed for 3 h at 37 °C in complete IMDM containing 5% FBS, followed by surface staining. A Zombie UV Fixable Viability Kit (BioLegend) or LIVE/DEAD Fixable Far Red Dead Cell Stain Kit (Thermo Fisher Scientific) was used to label and exclude dead cells from analysis. The complete list of antibodies used in this study is shown in Table S1. Multiparametric flow cytometry was performed on LSRFortessa flow cytometers (from BD Biosciences) and analyzed with FlowJo v10.5 (Tree Star Inc., Ashland, OR, USA).

### Statistical analysis

Data analysis was performed using SigmaPlot 14 (Systat Software, Germany) or GraphPad Prism 8 (GraphPad Software, La Jolla, CA, USA). Parametric comparisons of normally distributed values that satisfied the variance criteria were made by unpaired Student’s *t* tests or by one-way analysis of variance (ANOVA). Data that did not pass the variance test were compared with nonparametric two-tailed Mann–Whitney rank sum tests or ANOVA on rank tests. The *p* values are indicated by asterisks as follows: *p < 0.05; **p < 0.01; ***p < 0.001.

## Supplementary information

Supplementary figures
