# Supplementary material for: Default polyfunctional T helper 1 response to ample signal 1 alone
Source: Cell Mol Immunol. 2020 Apr 20;18(7):1809–22. doi: 10.1038/s41423-020-0415-x (PMC8245500; doi:10.1038/s41423-020-0415-x)
Supplement: Supplementary file 1 — Supplementary figures [file 41423_2020_415_MOESM1_ESM.pdf]

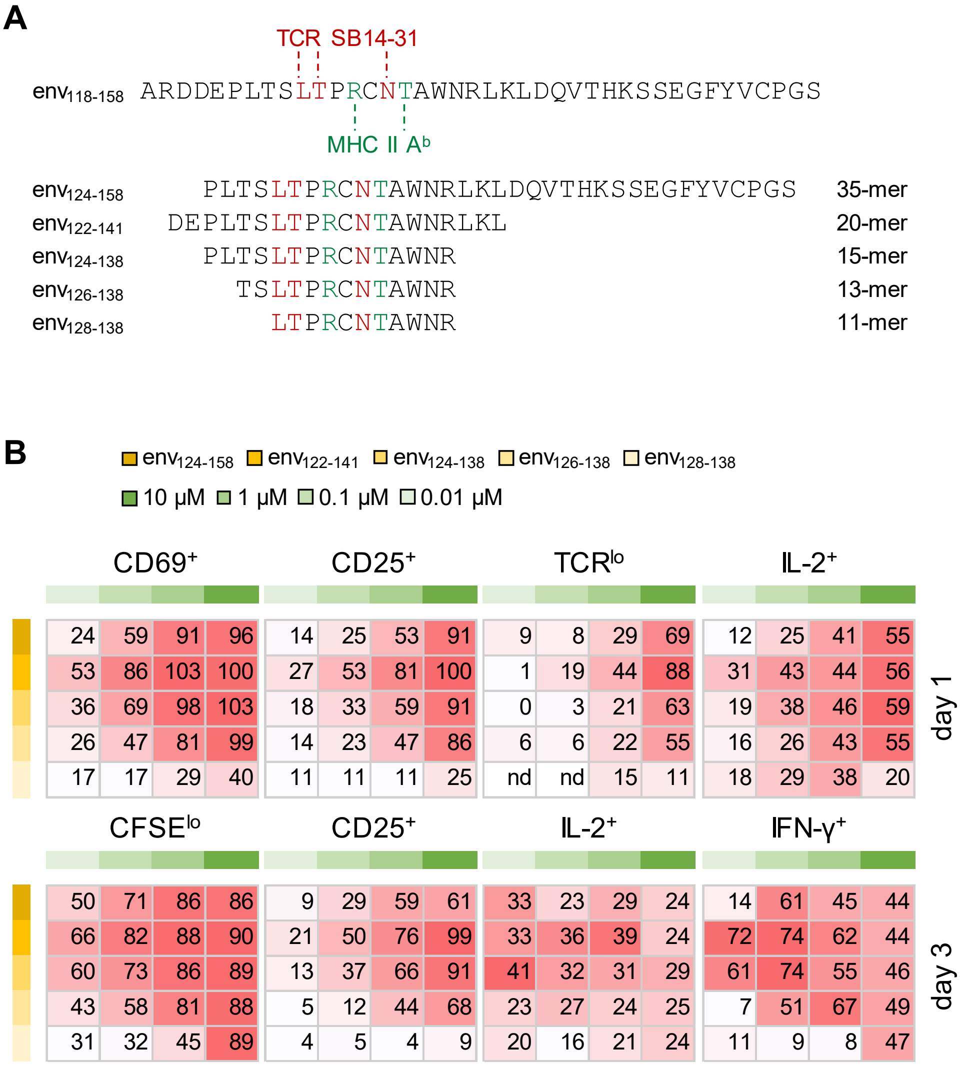

Figure S1. Assessing the optimal antigenic peptide length and dose. (**A**) Sequence of F-MLV $env_{118\text{-}158}$ depicting contact residues for MHC II A^b (green) and the SB14-31 TCR (red). The sequences of individual peptides of different length spanning the core epitope that were used for T cell stimulation is also shown. (**B**) Heatmaps of the response of EVα2 TCRαβ-transgenic $CD4^+$ T cells to stimulation with the indicated peptide length and dose. Data are pooled from 2-3 independent experiments.

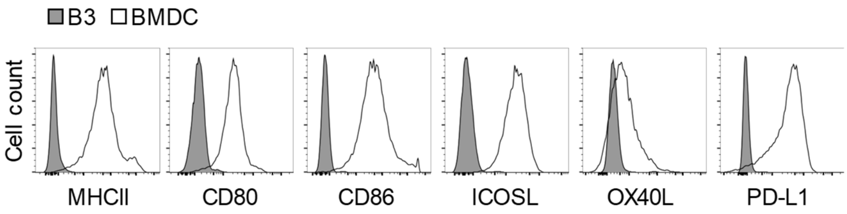

Figure S2. Lack of MHC II and co-stimulatory molecule expression in B3 cells. Representative flow cytometry histograms of surface expression of the indicated molecules in B3 cells. *Ex vivo* differentiated bone marrow-derived dendritic cells (BMDCs) are shown as a positive control.

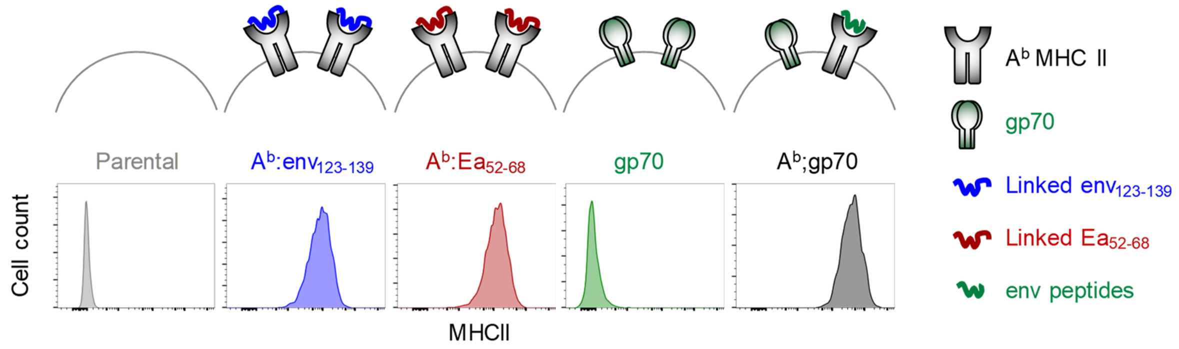

Figure S3. Expression of pMHC constructs in B3 cells. MHC II expression in parental B3 cells and those transduced with retroviral vectors expressing H2-A^b covalently linked to the 17-mer $env_{123-139}$ peptide (B3-A^b:$env_{123-139}$) or a control H2-E^k α chain peptide (B3-A^b:Ea_{52-68}). B3 cells transduced to express F-MLV gp70 alone (B3-gp70) or together with empty H2-A^b (B3-A^b;gp70) are also shown.

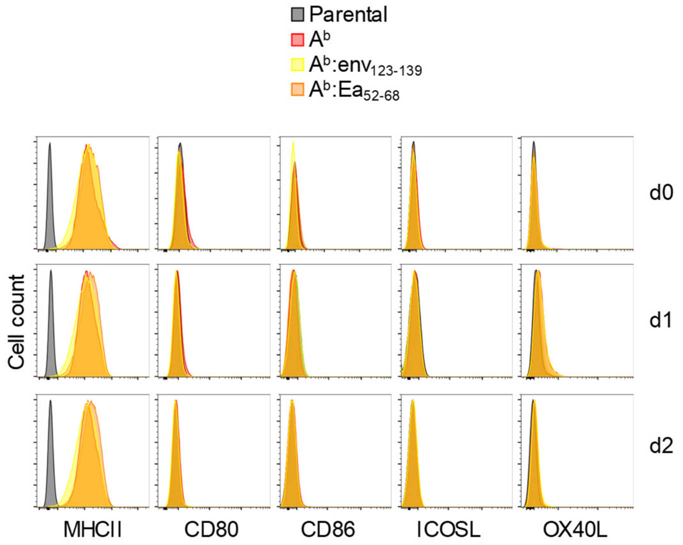

Figure S4. Lack of MHC II and co-stimulatory molecule expression in B3 cells after interaction with T cells. Representative flow cytometry histograms of surface expression of the indicated molecules in parental B3 cells and those transduced with retroviral vectors expressing empty H2-A^b (A^b) or covalently linked to the 17-mer $env_{123\text{-}139}$ peptide (B3-A^b:$env_{123\text{-}139}$) or a control H2-E^k α chain peptide (B3-A^b:$Ea_{52\text{-}68}$). B3 cells were co-cultured with EVα2 TCRαβ-transgenic $CD4^+$ T cells at 1:1 ratio and analysed over three consecutive days (days 0-2). One representative of two experiments is shown.

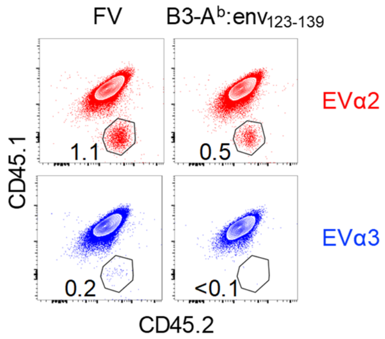

Figure S5. ***In vivo*** **priming of high, but not low affinity CD4$^+$ T cells by B3-A^b:env$_{123\text{-}139}$ cells.** EVα2 or EVα3 TCRαβ-transgenic CD45.2$^+$ CD4$^+$ T cells were adoptively transferred into CD45.1$^+$ CD45.2$^+$ WT recipients infected with FV (n=3) or immunised with B3-A^b:env$_{123\text{-}139}$ cells (n=3). Contour plots show the proportion of donor EVα2 or EVα3 T cells in gated CD4$^+$ T cells in each condition.

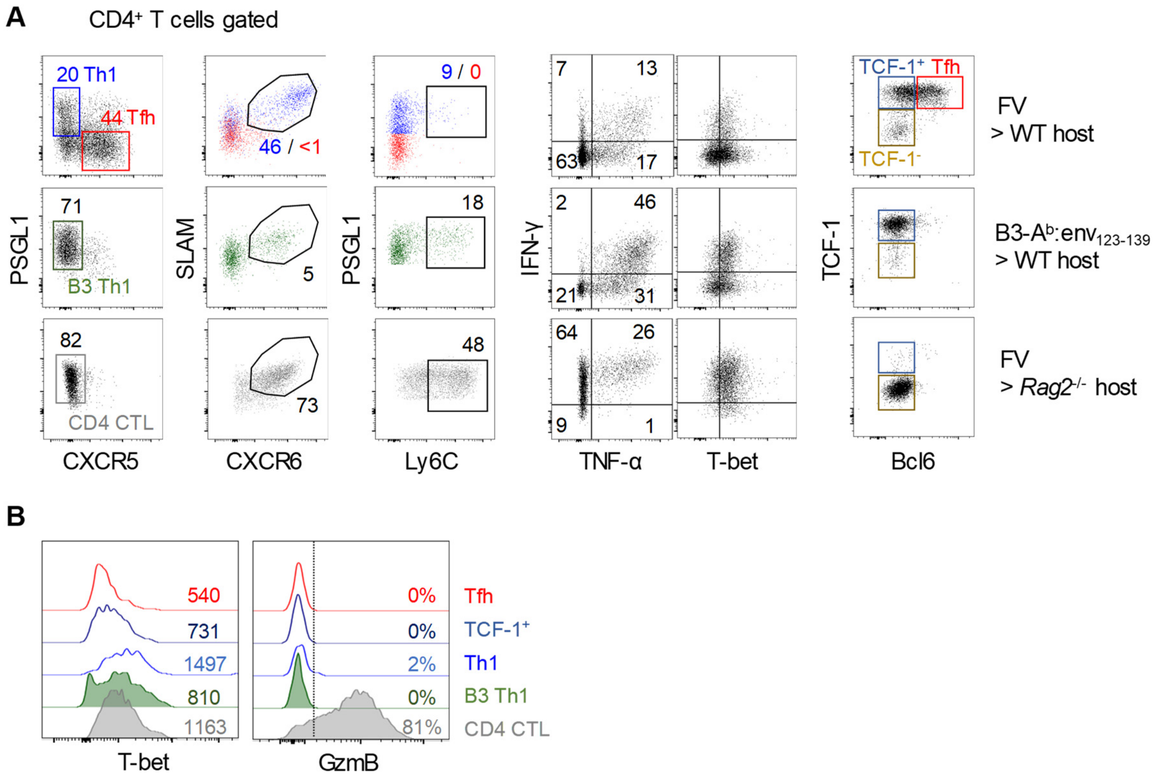

Figure S6. Th1, but not CTL differentiation of env-specific CD4$^+$ T cells primed by B3-A^b:env$_{123\text{-}139}$ cells. EF4.1 CD4$^+$ T cells were transferred into either WT mice that were immunized with B3-A^b:env$_{123\text{-}139}$ cells (n=4) or infected with FV (n=4), or into lymphopenic *Rag2*$^{-/-}$ hosts infected with FV (n=4). The phenotype of donor T cells was examined 7 days later. (**A**) Expression of the indicated Th1 or Tfh associated markers in the gated donor CD4$^+$ T cells and gates used for the definition of the Th subsets: Tfh, Tfh cells primed by FV; Th1, Th1 cells primed by FV; TCF-1$^+$, TCF-1$^+$ Th1 cells primed by FV; B3 Th1, Th1 cells primed by B3-A^b:env$_{123\text{-}139}$ cells; CD4 CTL, Th1 cells primed in *Rag2*$^{-/-}$ hosts. (**B**) Expression of T-bet and Granzyme B (GzmB), in the Th subsets as defined in (A). Numbers within the histograms denote the median fluorescence intensity (T-Bet) or the percentage of positive cells (GzmB).

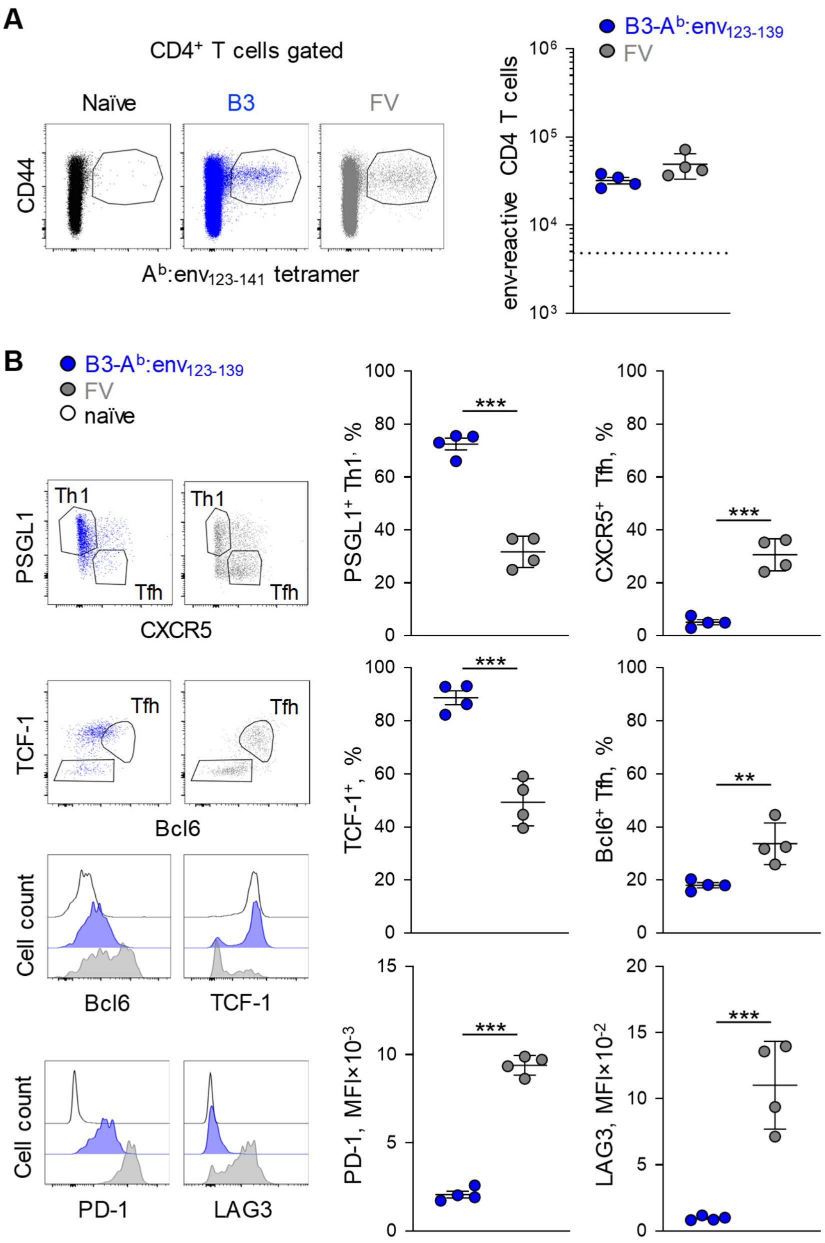

Figure S7. Priming of fully polyclonal non-transgenic env-specific CD4$^+$ T cells by B3-A^b:env$_{123\text{-}139}$ cells. WT mice were immunized with B3-A^b:env$_{123\text{-}139}$ cells (n=4) or infected with FV (n=4) and compared with naïve WT mice. Splenic env-reactive CD4$^+$ T cells were analysed by flow cytometry 7 days after infection or immunisation. (**A**) A^b:env$_{123\text{-}141}$-tetramer staining and absolute numbers (±SEM) of env$_{123\text{-}141}$-reactive CD44$^+$ CD4$^+$ T cells in these mice. (**B**) Expression of PSGL1, CXCR5, TCF-1, Bcl6, PD-1 and LAG3 in A^b:env$_{123\text{-}141}$-tetramer$^+$ CD44$^+$ CD4$^+$ T cells.

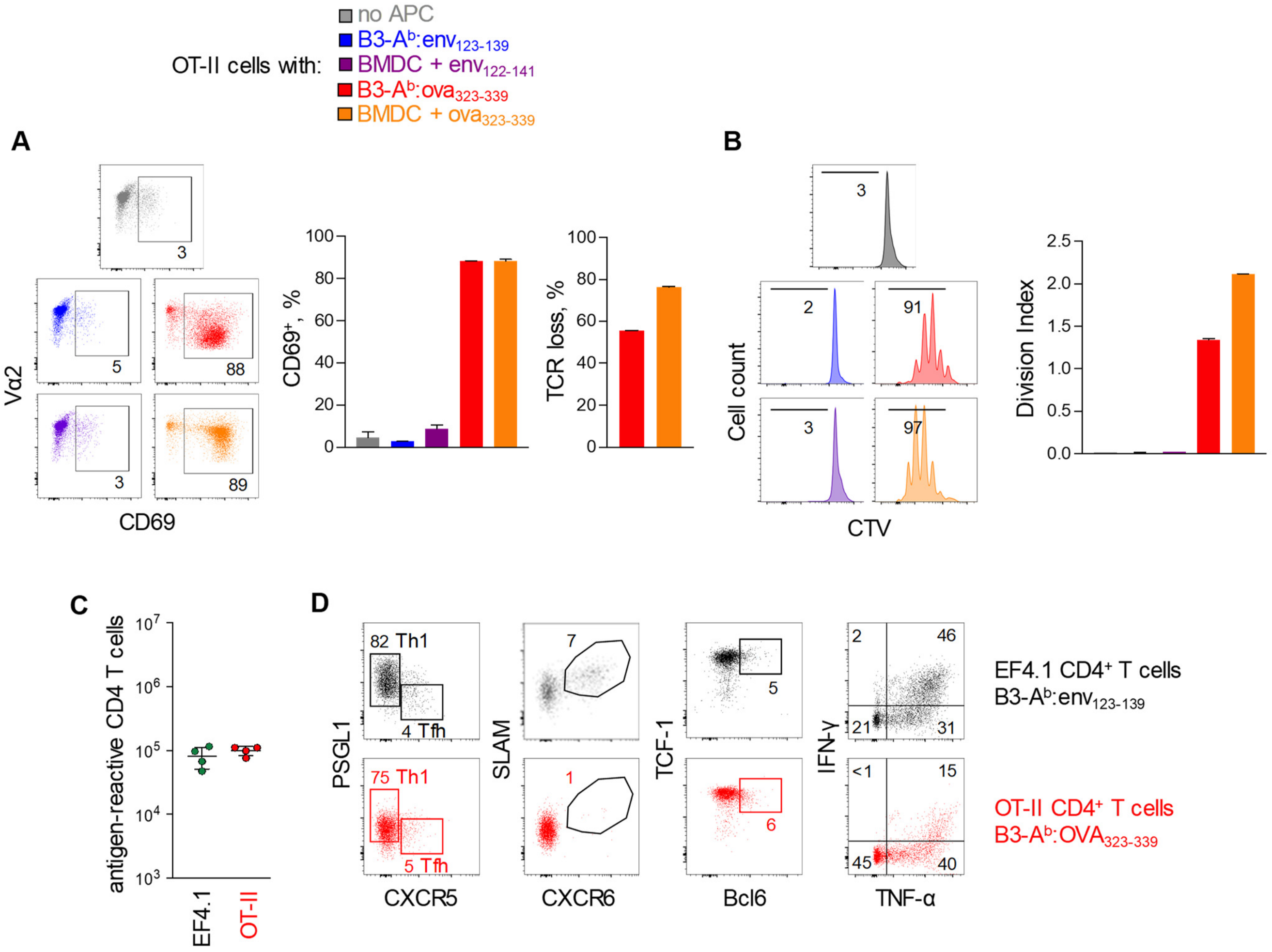

Figure S8. ***In vitro*** **priming and** ***in vivo*** **induction of Th1 phenotype in OT-II T cells by B3-A^b:ova$_{323-339}$ cells.** (**A**) CD69 and TCR Vα2 expression (*left*) and mean frequency (±SEM) (*right*) of primary OT-II TCRαβ-transgenic CD4$^+$ T cells upregulating CD69 or downregulating TCR Vα2 24 hrs after *in vitro* stimulation with the indicated APC. (**B**) CTV dilution (*left*) and calculated division index (±SEM) (*right*) of primary OT-II TCRαβ-transgenic CD4$^+$ T cells 72 hrs after *in vitro* stimulation with the same APCs as in (A). (**C**) Absolute numbers of CD44^{hi} donor TCRβ-transgenic EF4.1 and OT-II CD4$^+$ T cells 7 days after transfer into WT recipients and immunisation with B3-A^b:env$_{123-139}$ and B3-A^b:ova$_{323-339}$ cells, respectively. Each symbol represents an individual mouse. (**D**) Th1 and Tfh balance, assessed by mutually exclusive expression of PSGL1 and CXCR5, and SLAM, CXCR, TCF-1 and Bcl6 expression, and cytokine production of EF4.1 and OT-II cells primed as in (C).

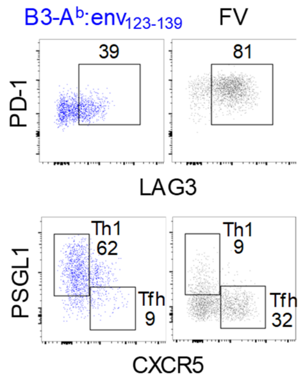

Figure S9. Induction of Th1 phenotype in monoclonal EVα2 T cells by B3-A^{b}:env$_{123-139}$ cells. EVα2 TCRαβ-transgenic CD45.2^{+} CD4^{+} T cells were adoptively transferred into CD45.1^{+} CD45.2^{+} WT recipients infected with FV (n=3) or immunised with B3-A^{b}:env$_{123-139}$ cells (n=3) and expression of PD-1, LAG3, PSGL1 and CXCR5 was examined in splenic donor T cells 7 days later.

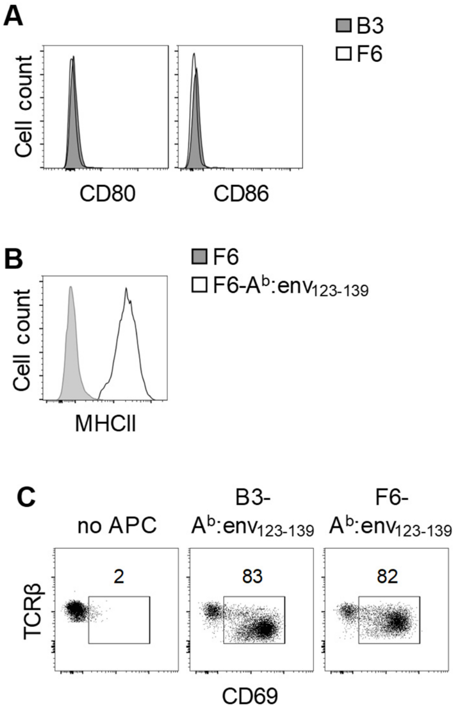

Figure S10. Phenotype and antigen presenting capacity of F6-A^b:env$_{123-139}$ cells. (**A**) CD80 and CD86 expression in parental F6 pro-B cell leukaemia cells, compared to B3 cells. (**B**) MHC II expression in F6 cells after transduction with an A^b:env$_{123-139}$-expressing vector, compared with the parental F6 cells. (**C**) EVα2 CD4$^+$ T cell activation (assessed by CD69 upregulation and TCR downregulation) after overnight culture alone or with F6-A^b:env$_{123-139}$ cells or B3-A^b:env$_{123-139}$ cells. Data are representative of 2-3 independent experiments.

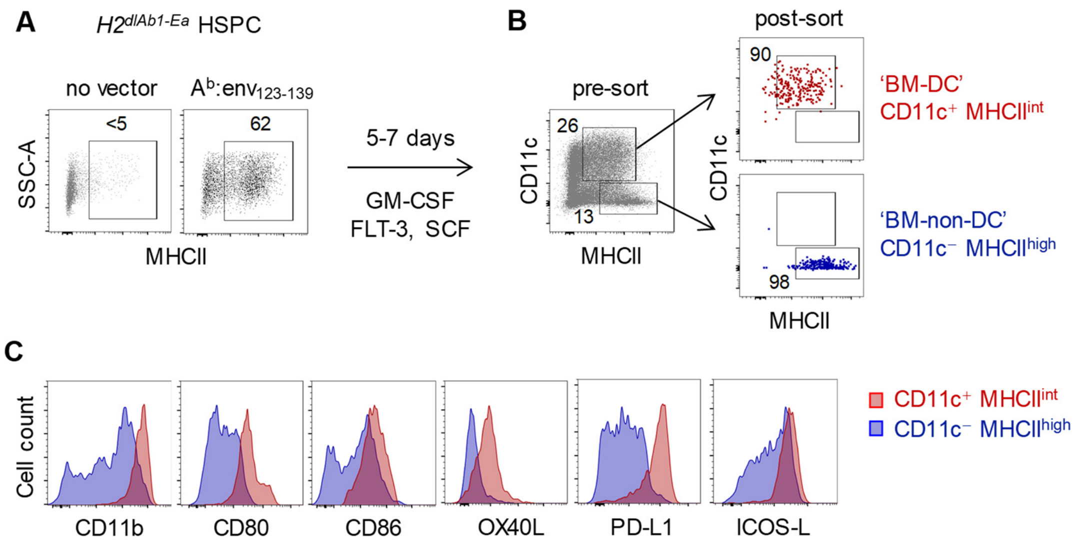

Figure S11. Generation and phenotype of bone marrow-derived CD11c^{+} DCs and CD11c^{-} non-DCs. Enriched hematopoietic stem and progenitors cells (HSPCs) from *H2$^{dlAb1-Ea}$* bone marrow cell suspensions were transduced with an A^{b}:env$_{123-139}$-expressing vector. 48 hrs after transduction, cells were seeded in DC differentiation media, containing GM-CSF, FLT3L and SCF for 5-7 days. Two subpopulations expressing MHC II, CD11c^{+} MHCII^{int} (BM-DC) and CD11c^{-} MHCII^{hi} (BM-non-DC), were purified by cell-sorting. (**A**) MHCII expression in HSPCs 48 hrs post-transduction. (**B**) CD11c and MHCII expression following 5-7 days of growth in DC differentiation media, before and post cell-sorting of the indicated populations. (**C**) Expression of the indicated co-stimulatory or co-inhibitory molecules in BM-DC and in BM-non-DC. Data are representative of at least 3 independent experiments.

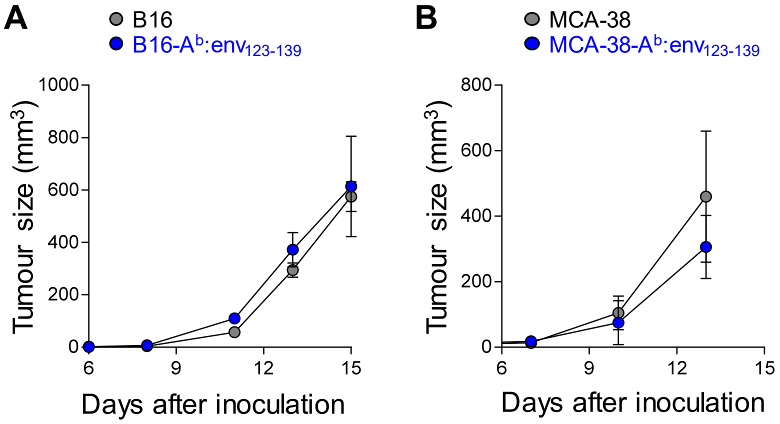

Figure S12. Amplified signal 1 in solid tumours does not promote rejection. B16 melanoma and MCA-38 colon adenocarcinoma cells were transduced with an A^b:$env_{123\text{-}139}$-expressing vector and compared for *in vivo* growth with the respective parental cells. (**A**) Mean growth (±SEM) of parental B16 and B16-A^b:$env_{123\text{-}139}$ cells in WT recipients (n=6 for each cell line). (**B**) Mean growth (±SEM) of parental MCA-38 and MCA-38-A^b:$env_{123\text{-}139}$ cells in WT recipients (n=6 for each cell line).

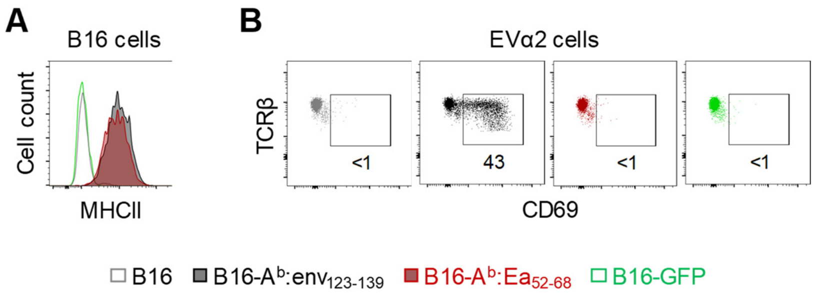

Figure S13. MHC II expression and antigen presenting capacity of B16-A^b:$env_{123\text{-}139}$ cells. (**A**) MHC II expression in parental B16 cells or those transduced with vectors expression A^b:$env_{123\text{-}139}$, A^b:$Ea_{52\text{-}68}$ or GFP. (**B**) EVα2 $CD4^+$ T cell activation (assessed by CD69 upregulation and TCR downregulation) after overnight culture alone or with B16-A^b:$env_{123\text{-}139}$, B16-A^b:$Ea_{52\text{-}68}$ or B16-GFP cells. Data are representative of 2 independent experiments.
